# An Optically Silent Epoxysilane Chemistry for Paper-Based ABO Blood Typing

**DOI:** 10.3390/bios16090468

**Published:** 2026-08-27

**Authors:** Chinnawut Pipatpanukul, Komkrisd Wongtimnoi, Laurent Mezeix, Santi Phosri

**Affiliations:** 1 Department of Advanced Materials Engineering, Faculty of Engineering, Burapha University, Chonburi 20131, Thailand; komkrisd@eng.buu.ac.th (K.W.); laurentm@eng.buu.ac.th (L.M.); 2 Department of Chemical Engineering, Burapha University, Chonburi 20131, Thailand; santi.ph@eng.buu.ac.th

**Keywords:** paper-based analytical device, ABO blood typing, antibody immobilisation, epoxysilane, glutaraldehyde chromophore, point-of-care diagnostics

## Abstract

Safe transfusion depends on rapid, accurate ABO typing, yet reference methods require centrifuges and instrumentation unavailable at the point of need. Here, a paper-based ABO-RhD typing device built on a covalent, optically silent surface chemistry is reported. Aminosilane (APTES) and epoxysilane (GPTMS) functionalisation of Whatman cellulose was compared by water contact angle, energy-dispersive X-ray spectroscopy (SEM-EDS) and infrared spectroscopy (FT-IR) across two paper grades, three silane concentrations (5, 10 and 20% *v*/*v*) and three reaction times (1–6 h). APTES produced a strongly hydrophobic layer that impeded aqueous wicking, and its glutaraldehyde activation generated a red-brick chromophore incompatible with a red-channel readout. GPTMS coupled antibodies in a single mild step, without a crosslinker or visible chromophore, while preserving wicking. GPTMS (10% *v*/*v*, 3 h, Whatman No. 4) with a six-cycle 100 µL saline wash was selected; antibodies were immobilised in a four-zone layout (anti-A, anti-B, anti-D and control) within a 3D-printed two-compartment housing that traps agglutinated cells while free cells wash through. On 80 EDTA clinical blood samples (20 each of groups A, B, AB and O) the device classified every sample correctly (accuracy 100%; 95% confidence interval 95.4–100%), with visual and instrumented reads in full agreement. All samples were RhD-positive, so the anti-D channel is validated here for the positive call.

## 1. Introduction

Correct identification of a recipient’s ABO blood group is one of the most safety-critical steps in transfusion medicine, because transfusion of ABO-incompatible blood can trigger acute intravascular haemolysis [1]. ABO typing is therefore repeated at several points along the transfusion chain at donation, on entry to the blood bank and again before issue and is performed a very large number of times each year [2]. The established laboratory methods (tube and slide haemagglutination, gel-card column agglutination and automated microplate systems) are accurate but depend on centrifuges, trained technologists and, in the automated formats, imported instrumentation with a substantial per-test cost. These requirements are difficult to meet in mobile donation drives, small or rural hospitals, emergency and field settings, and low-resource health systems, where point-of-care diagnostics that are rapid, low-cost and readable without specialist expertise are increasingly recognised as an unmet need [3].

Cellulose paper is an attractive substrate for this class of test. It is inexpensive, abundant and disposable, wicks aqueous samples by capillary action, and its porous fibre network can physically trap agglutinated red blood cells while allowing non-agglutinated cells to wash through a size-exclusion mechanism well suited to blood-group readout [4,5]. Since the foundational paper-microfluidics work of the Whitesides group [6], microfluidic paper-based analytical devices (µPADs) have matured into a broad point-of-care platform [7], and blood grouping has become one of their most-studied clinical applications [4]. Building on this, Khan and co-workers introduced an instantaneous bioactive-paper blood diagnostic [8], Al-Tamimi and co-workers validated a paper assay against ABO and RhD reference results [9], and Li and co-workers reported a device that prints the blood type “in writing” [10]. Noiphung and co-workers combined simultaneous forward-and-reverse ABO with Rh typing on a single device [11], and Songjaroen and co-workers added barcode-style interpretation [12]. More recently the field has moved toward clinical deployment and richer antigen coverage. Chomean and co-workers combined paper devices with portable and image-based readout to phenotype ABO, RhD and minor Rh antigens [13], Larpant and co-workers added deep-learning interpretation of five Rh antigens [14], Chang and co-workers integrated forward typing with haemophilia screening in a portable reader [15], and Al-Tamimi and co-workers extended the paper format to direct serum and plasma separation [16]. Recent overviews of paper- and microfluidics-based blood typing document this rapid progress [17]. Our own group previously demonstrated ABO-Rh (D) typing on a chemically modified poly (methyl methacrylate) surface, establishing the epoxysilane (GPTMS) coupling route used here [18].

Despite this progress, turning a paper assay into a robust, field-deployable device requires three engineering problems to be solved together rather than in isolation. The first is the antibody-surface chemistry, antibodies must be attached covalently and stably, with preserved antigen-binding activity and critically for a colorimetric readout without introducing any colour of their own [19]. The second is fluid management. The wash that removes non-agglutinated cells from negative zones must give a wide, reproducible positive-versus-negative contrast within a self-contained device rather than by manual pipetting on an open strip. The third is validation on real blood against a reference standard. Prior reports commonly treat the surface chemistry as a solved, off-the-shelf step typically the aminosilane (APTES) plus glutaraldehyde Schiff-base route inherited from silica-bead and biosensor immunoassays [19], even though epoxysilane (GPTMS) chemistry offers direct, single-step covalent coupling of antibody amines and has been used for stable antibody immobilisation on silica and electrode surfaces [20].

In this work, APTES and GPTMS functionalisation of Whatman cellulose are compared by water contact angle, SEM-EDS and FT-IR. The APTES–glutaraldehyde chromogenic side reaction that disqualifies the conventional route is documented and GPTMS is identified as the working substrate on the combined basis of positive-versus-negative signal contrast and PBS wash-through time. A single-step GPTMS epoxide–amine antibody immobilisation and a six-cycle 100 µL PBS wash protocol are established, the functionalised paper is integrated into a 3D-printed two-compartment housing that manages the wash fluid vertically, and the complete device is validated on 80 blood samples against the reference typing result. The outcome is reported fully, together with the practical storage limitation of the antibody-loaded insert, to give an honest, end-to-end account of a covalent, optically clean, instrument-light paper device for ABO blood typing. For every sample, each zone was scored as positive or negative by background subtraction against the control-channel signal, giving an unambiguous four-zone pattern; on this basis, all 80 device calls matched the reference.

## 2. Materials and Methods

### 2.1. Materials, Antibodies and Blood Samples

Whatman cellulose filter papers of Grade No. 1 (nominal pore size 11 µm) and No. 4 (20–25 µm) (Cytiva, Marlborough, MA, USA) were used as substrates. The silane coupling agents 3-aminopropyltriethoxysilane (APTES) and 3-glycidyloxypropyltrimethoxysilane (GPTMS) were obtained from Dow Corning (Thailand) Co., Ltd. (Bangkok, Thailand). Glutaraldehyde (GA) and phosphate-buffered saline (PBS) were obtained from Sigma-Aldrich (St. Louis, MO, USA); methanol, ethanol and hydrochloric acid were obtained from RCI Labscan (Bangkok, Thailand). Ethanolamine and glycine were of analytical grade. Deionised (DI) water was used throughout. The two filter paper grades were selected: No. 1 (11 µm) and No. 4 (20–25 µm), which permit agglutinate trapping with rapid buffer wash-through (manufacturer specifications [21]). Deionised water (resistivity: 18.2 MΩ·cm) was produced using a Milli-Q EQ 7000 system (Merck Millipore, Molsheim, France; Type 1 ultrapure water) and used throughout.

Monoclonal anti-A, anti-B and anti-D (Rh) typing reagents, together with standard reagent ABO red blood cells (groups A, B and O), were obtained from the National Blood Centre, Thai Red Cross Society (Bangkok, Thailand). The antibody stock concentration was determined using a NanoDrop One spectrophotometer (Thermo Fisher Scientific, Waltham, MA, USA) at 280 nm, and the stock was diluted with PBS (pH 7.4) to a final working concentration of 0.62 mg mL^−1^, which was used for both surface functionalisation and validation experiments.

For device validation, EDTA-anticoagulated blood samples were obtained from the Blood Bank, Chonburi Hospital (Chonburi, Thailand). All samples were leftover specimens remaining after the completion of routine diagnostic testing; no blood was drawn from any patient for the purposes of this study. Approximately 200 such specimens were collected continuously over the course of the study, of which 80 (20 each of groups A, B, AB and O) were tested; the reference ABO-RhD group of each sample was provided by the hospital. This study was approved by the Institutional Review Board of Burapha University (protocol code Sci 071-2561). The specimens were irreversibly de-identified before transfer and carried no identifiers or clinical information other than the reference ABO-RhD group, so that no sample could be traced back to an individual patient by the investigators or by any other party. For testing, red blood cells (RBCs) were separated from plasma, washed with normal saline, stored in Alsever’s solution at 4–8 °C, and washed with PBS immediately before assay. The RBC of 10% (*v*/*v*) suspension in PBS was used for the functional assays. Reconstituted reagent-cell suspensions and EDTA whole blood were compared to confirm compatibility across sample formats. The 80 specimens were selected from the approximately 200 available by simple random draw, stratified to 20 per ABO group (A, B, AB and O). Because the only attribute linked to each leftover specimen was its ABO-RhD group, this randomised, group-balanced selection formed part of the de-identification safeguard, ensuring that the tested subset could not be traced back to individual donors. The reference ABO-RhD group of every sample was independently confirmed by conventional slide agglutination, in addition to the hospital tube label, and this served as the reference standard against which the device result was compared.

### 2.2. Silane Functionalisation of Cellulose Paper

Whatman filter papers (No. 1 and No. 4) were cut into 5 × 5 cm^2^ sheets, rinsed with methanol to remove surface contaminants and air-dried prior to surface modification. Silanisation was performed using either APTES or GPTMS; for both silanes, each of the two paper grades was functionalised at every combination of three concentrations (5, 10 and 20% *v*/*v*) and three reaction times (1, 3 and 6 h), yielding a full factorial of 18 conditions per silane. APTES (aminosilane) and GPTMS (epoxysilane) were chosen as the two most widely used silane coupling agents for antibody attachment to hydroxyl-bearing substrates, representing the amine/glutaraldehyde and the epoxide routes, respectively [19,20,22,23]. The concentration (5–20% *v*/*v*) and reaction-time (1–6 h) windows were set from previously reported silanisation conditions for cellulose and silica surfaces [22,23] and refined by preliminary screening, so that the grid spans the onset, rise and plateau of surface grafting.

The APTES solution was prepared in ethanol and stirred for 15 min before use. The paper substrates were immersed in the solution for the prescribed reaction time in a chemical fume hood, rinsed thoroughly with DI water (three to five times), dried under nitrogen gas and thermally cured at 100 °C for 4 h to promote siloxane-network formation and covalent bonding with the cellulose hydroxyl groups. The functionalised papers were then stored in a desiccator until use.

The GPTMS solution was prepared in DI water acidified to pH 3 with 1 M HCl and stirred for 15 min before immersion. The paper substrates were immersed under identical conditions in a chemical fume hood, rinsed with DI water (three to five times) and dried under nitrogen gas. The substrates were then thermally cured at 80 °C for 4 h to complete silane condensation and form the epoxy-functional surface, before being stored in a desiccator until further use.

### 2.3. Surface Characterisation

Water wettability was evaluated using a contact-angle goniometer (OCA; DataPhysics Instruments GmbH, Filderstadt, Germany). A 5 µL droplet of deionised water was gently deposited onto each modified paper surface and the droplet profile recorded immediately after dispensing. Static water contact angles were determined using the instrument analysis software. Five independent measurements (*n* = 5) were performed at randomly selected locations on each sample, and the results are reported as the mean ± standard deviation.

Surface morphology, fibre structure, pore dimensions and elemental silicon content were characterised using a LEO 1450 VP scanning electron microscope (Carl Zeiss, Oberkochen, Germany) equipped with an energy-dispersive X-ray spectroscopy (EDS) detector. Samples were sputter-coated with a thin layer of gold prior to imaging to minimise surface charging. Images were acquired at an accelerating voltage of 15 kV, with the corresponding scale bars provided in each micrograph. The silicon content was quantified from three independent EDS spectra collected from different regions of each sample, and the mean silicon weight percentage (wt%) was used as a quantitative indicator of silane grafting.

Chemical functional groups were analysed using a Nicolet 6700 Fourier-transform infrared spectrometer (Thermo Fisher Scientific, Waltham, MA, USA) operated in attenuated total reflectance (ATR) mode. Spectra were collected over the range 4000–650 cm^−1^ at a spectral resolution of 1 cm^−1^ with 64 accumulated scans per spectrum. The characteristic absorption bands of Si–O–Si (~1050 cm^−1^), Si–O–C (~780 cm^−1^) and the primary-amine N–H bending vibration (~1630 cm^−1^) were used to confirm silane grafting. For GPTMS-functionalised samples, the epoxy-ring vibration (~910 cm^−1^) was evaluated only when experimentally observed in the recorded spectra.

### 2.4. Antibody Immobilisation

APTES-functionalised paper substrates were activated by immersion in 2.5% (*v*/*v*) glutaraldehyde solution for 2 h at room temperature (approximately 25 °C) to generate reactive aldehyde groups for Schiff-base coupling, then rinsed thoroughly with PBS to remove unreacted glutaraldehyde. Antibody solution (0.62 mg mL^−1^) was dispensed onto the activated surface and incubated for 30 min at room temperature (approximately 25 °C), after which the substrates were washed several times with PBS to remove unbound antibody. The immobilised antibodies were evaluated with a 10% (*v*/*v*) RBC suspension in PBS; this route served as the comparison method for assessing the colour interference associated with the APTES–glutaraldehyde chemistry.

GPTMS-functionalised paper substrates were functionalised directly by dispensing antibody solution (0.62 mg mL^−1^) prepared in carbonate buffer (pH 9.6) onto the paper surface. The substrates were incubated for 30 min at room temperature to allow covalent coupling between the epoxy groups of GPTMS and the antibody amino groups, then washed several times with PBS to remove unbound antibody. The immobilised antibodies were evaluated with a 10% (*v*/*v*) RBC suspension in PBS. For the four-zone detection device, anti-A, anti-B, anti-D and a PBS (antibody-free) control were immobilised in separate reaction zones defined by an H-shaped silicone barrier patterned on the paper substrate.

### 2.5. Imaging, Quantification and Statistical Analysis

The washed paper devices were imaged using a Basler ace acA1920-155uc colour CMOS camera (Basler AG, Ahrensburg, Germany) equipped with a Computar M5018-MP2 machine-vision lens (Computar, CBC Co., Ltd., Tokyo, Japan). Images were acquired under constant illumination and a fixed imaging geometry throughout the study. Image analysis was performed using ImageJ (v1.54; National Institutes of Health, Bethesda, MD, USA). For each reaction zone, a circular region of interest (ROI) was defined, the red channel was isolated, and the integrated density (IntDen; arbitrary units) was extracted. Two signal metrics were used for data analysis. For clinical interpretation, the IntDen of each antibody zone was background-corrected against the control zone measured on the same device. The net integrated density was calculated as

(1) IntDen_net_ = IntDen_zone_ − IntDen_control_

where IntDen_zone_ and IntDen_control_ represent the integrated densities of the antibody and control zones, respectively. This correction was applied to account for device-to-device and illumination-related background variation. A reaction zone was classified as positive when IntDen_net_ exceeded the predefined threshold and as negative otherwise.

For condition optimisation, the IntDen of each reaction zone was normalised to the maximum IntDen of the corresponding response-surface map:

(2) I_norm_ = IntDen_zone_/IntDen_max_

where I_norm_ represents the normalised intensity and IntDen_max_ is the maximum IntDen of the corresponding response-surface map. The effects of silane concentration and reaction time were visualised as three-dimensional response surfaces to identify the condition providing the greatest separation between positive and negative agglutination signals. Accordingly, clinical readouts are reported as net IntDen, whereas optimisation response surfaces are presented as normalised intensity throughout the text and figures.

For clinical validation, the positive/negative patterns obtained from the anti-A, anti-B, and anti-D reaction zones were interpreted according to standard blood-grouping criteria and compared with the corresponding reference results. Diagnostic accuracy and group-specific sensitivity were calculated, and the corresponding 95% confidence intervals (CIs) were estimated using the Wilson method.

## 3. Results and Discussion

### 3.1. Silane Functionalisation and Surface Characterisation

**Figure 1:**
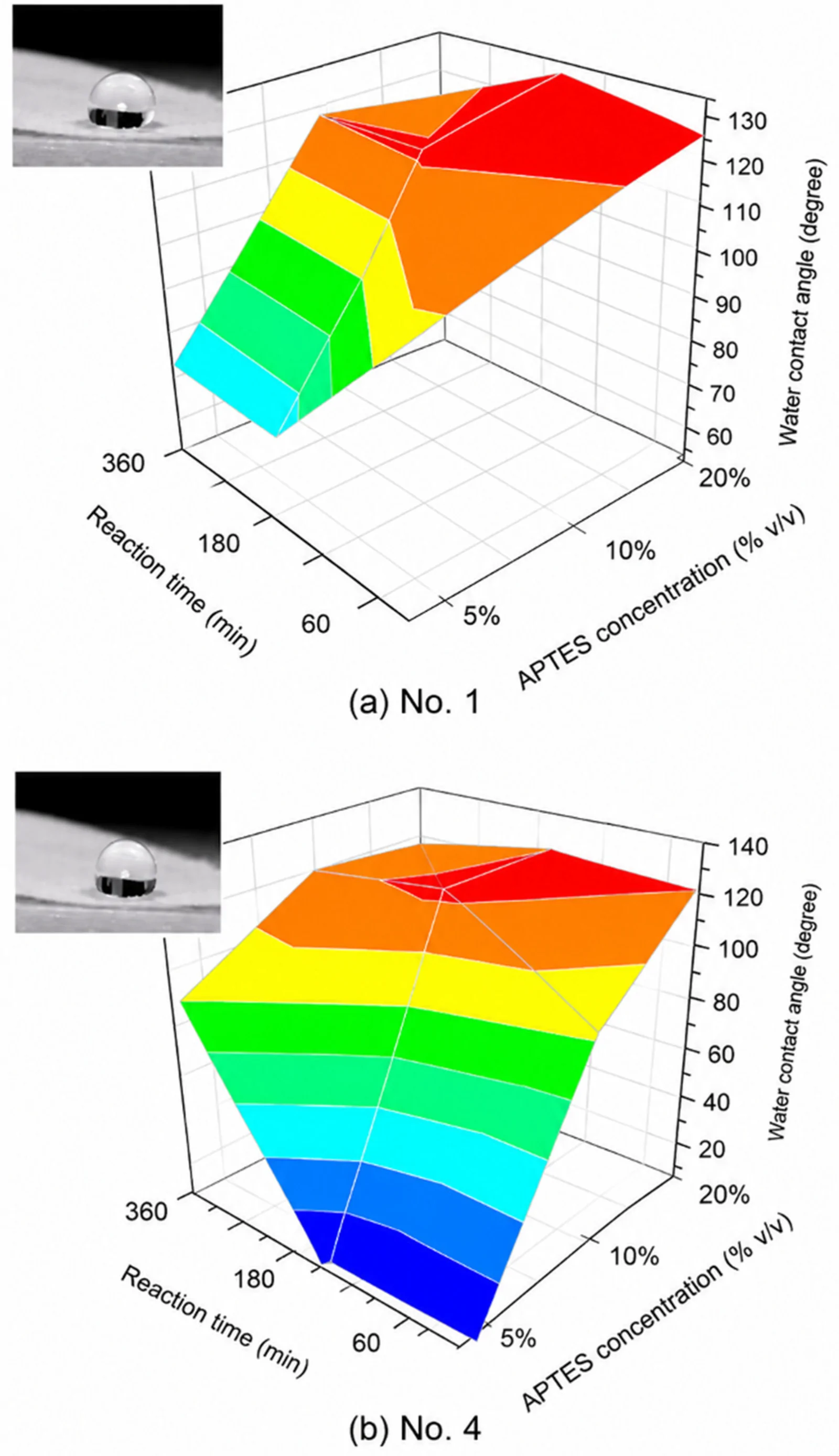
Water contact angle (measured 5 min after droplet deposition) of APTES-functionalised paper, plotted as a response surface against APTES concentration (% *v*/*v*) and reaction time (min) for Whatman (**a**) No. 1 and (**b**) No. 4. The angle rises with both concentration and reaction time, reaching approximately 120°. Colours indicate the water contact angle value, from blue (low, more hydrophilic) to red (high, most hydrophobic), as shown in the adjacent colour bar.

Water contact angle (WCA), measured 5 min after droplet deposition, revealed opposite and diagnostically useful behaviour for the two silanes and gave a direct, quantitative confirmation of the change in surface wettability produced by the treatment. Untreated Whatman cellulose is intrinsically superhydrophilic. A water droplet is imbibed by the fibre network within about one second, so that no stable sessile angle can be defined (effective WCA ≈ 0°). After APTES functionalisation the WCA rose systematically and monotonically with both silane concentration and reaction time and was mapped as a response surface for each paper grade (Figure 1), increasing from this near-zero baseline to approximately 120° at the higher concentrations and longer reaction times before levelling off a change in more than 120° that converts an absorbent substrate into a water-repellent one.

Two features indicate that this reflects genuine covalent grafting rather than a transient surface effect. First, the dose-dependent, plateauing rise is the signature of progressively increasing silane coverage in agreement with the surface silicon content measured by EDS (Section 3.1). Second, the effect was temporally stable. On the APTES-functionalised surface, the droplet retained a contact angle close to 120° for at least 30 min without spreading or being absorbed (Figure S1), whereas a physisorbed or unstable layer would relax within seconds. A large, persistent increase in WCA of this kind is the accepted fingerprint of successful hydrophobic silanisation of cellulose. For the GPTMS-functionalised surface, in contrast, the droplet spread rapidly and reached complete wetting (0°) within 5 min on both paper grades (Figure S2), so no stable sessile angle could be defined and the angle could not be tracked as a function of the synthesis conditions; the epoxide-terminated layer is hydrophilic and preserves the capillary wicking of the paper. This difference follows from the underlying surface chemistry: the amine-terminated propyl chain of APTES yields a comparatively non-polar, water-repellent surface, whereas the more polar, ring-opened GPTMS layer remains wettable. Although the persistent hydrophobicity of the APTES surface is itself direct evidence of effective grafting, it is a liability for a paper-based device because it impedes the lateral flow and PBS washing on which the assay depends. This constitutes the first indication that the two chemistries are not interchangeable for paper-based blood typing.

**Figure 2:**
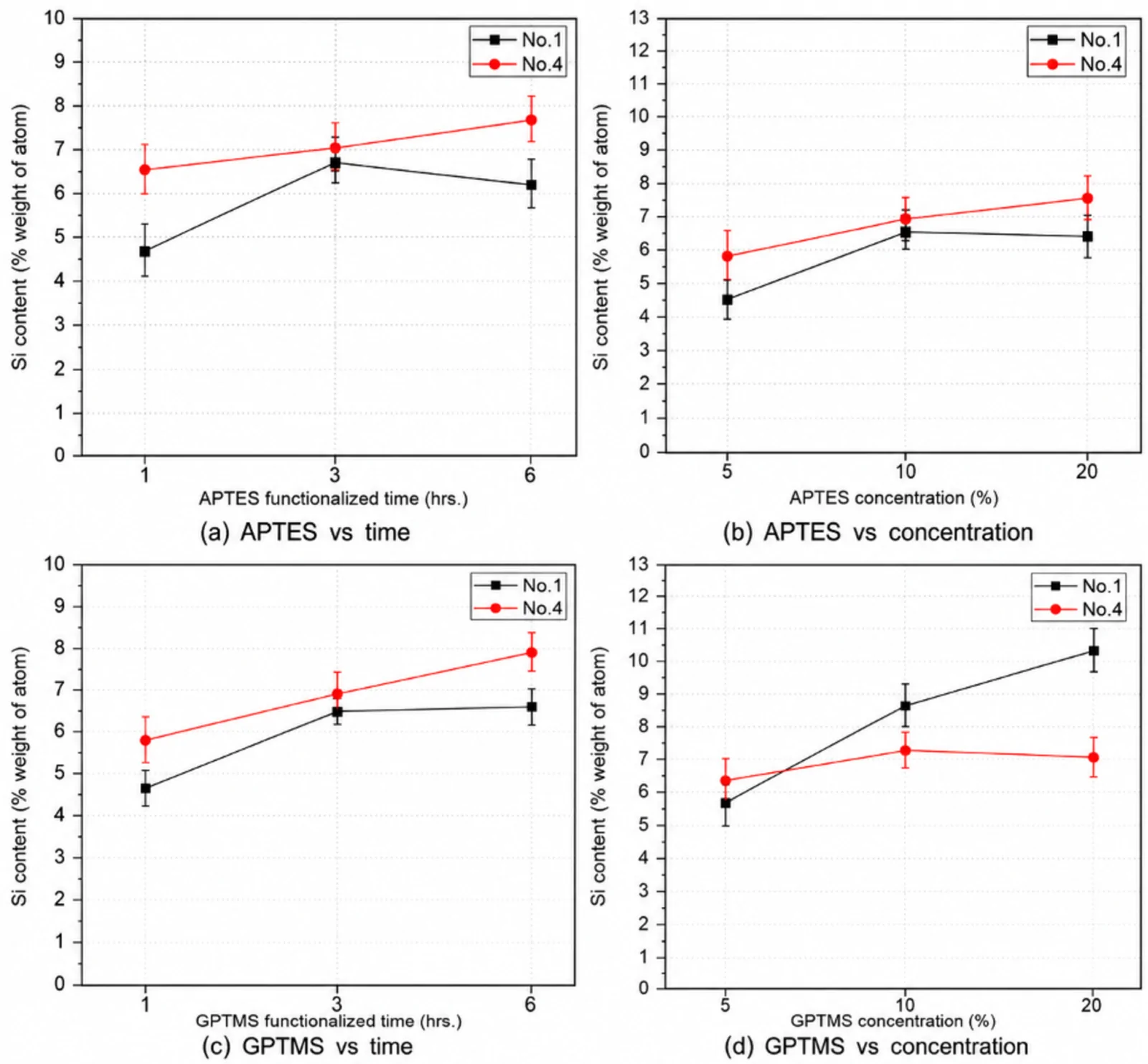
Silicon content (wt%) determined by SEM–EDS for Whatman No. 1 and No. 4: (**a**) APTES versus reaction time at 10% *v*/*v*; (**b**) APTES versus concentration at 3 h; (**c**) GPTMS versus reaction time at 10% *v*/*v*; (**d**) GPTMS versus concentration at 3 h. Both silanes graft efficiently, giving silicon contents of approximately 5–10 wt% across the conditions examined; the highest value was recorded for GPTMS on Whatman No. 1 at 20% *v*/*v*. On Whatman No. 4, the grade used for the device, the silicon content saturates above 10% *v*/*v*. Error bars denote the standard deviation of three EDS spectra.

The silicon content determined by SEM-EDS confirmed successful grafting for both silanes (Figure 2; representative micrographs, spectra and elemental tables are provided in Figures S3 and S4). Under the working condition subsequently adopted for the device (10% *v*/*v*, 3 h), the surface silicon content was 4.98 wt% for APTES and 7.05 wt% for GPTMS on Whatman No. 1, and 5.88 wt% and 8.46 wt%, respectively, on Whatman No. 4 (Figures S3 and S4). The APTES spectra additionally show a nitrogen signal from the aminopropyl group, accounting for the balance of the composition, whereas the GPTMS spectra contain only carbon, oxygen and silicon, as expected for an epoxysilane. Both silanes therefore graft efficiently onto cellulose, and under identical conditions the two silanes give comparable silicon coverage, with GPTMS reaching the higher maximum across the parameter space examined. Across the wider parameter space (Figure 2), the silicon content of both silanes increased between 1 and 3 h and changed comparatively little thereafter, and rose with concentration up to about 10% *v*/*v* before levelling off on Whatman No. 4; on Whatman No. 1 the GPTMS loading continued to increase to approximately 10 wt% at 20% *v*/*v*, the highest value recorded in this study. The results implied that the silicon content on Whatman No. 1 and 4 saturates above 10% *v*/*v*, increasing the silane concentration further raises reagent cost without increasing grafting. APTES and GPTMS at 10% *v*/*v* and 3 h were therefore adopted for all subsequent work.

**Figure 3:**
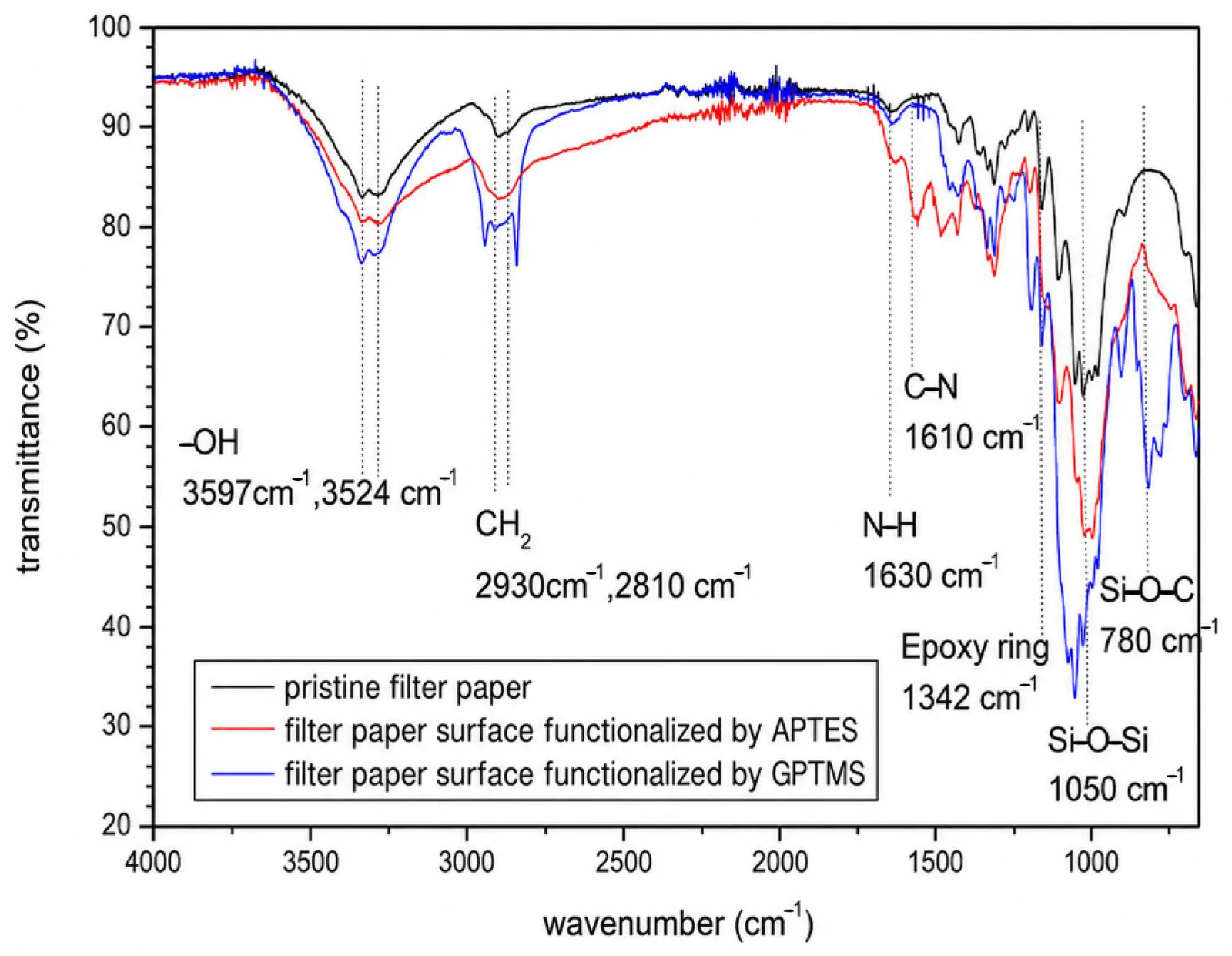
ATR-FTIR of pristine, APTES- and GPTMS-functionalised Whatman No. 4 (10% *v*/*v*, 3 h). Both silanes show Si–O–Si (1050 cm^−1^) and Si–O–C (780 cm^−1^); the 1630 cm^−1^ N–H band is APTES-specific.

The FT-IR spectra of pristine, APTES- and GPTMS-functionalised Whatman No. 4 (10% *v*/*v*, 3 h) displayed, for both silanes, the Si–O–Si asymmetric stretching band at 1050 cm^−1^ and the Si–O–C linkage to cellulose at 780 cm^−1^, confirming covalent condensation with the surface hydroxyl groups. The APTES spectrum additionally displayed the primary-amine N–H bending band at 1630 cm^−1^, the molecular signature of the aminopropyl side chain, which was absent from the GPTMS spectrum. These assignments corroborate the wettability and EDS data, indicating that both silanes graft to the cellulose while presenting distinct terminal functional groups (Figure 3).

Pore geometry governed the choice of paper grade (Figure S5). Whatman No. 1 (11 µm) has pores only marginally larger than the 7.2–8.4 µm diameter of a red blood cell, so that even non-agglutinated cells tend to be retained and cannot be washed clear, blurring the distinction between positive and negative zones. Whatman No. 4 (20 µm) possesses pores appreciably larger than a single red cell, so that agglutinated clusters are retained on antibody-bearing zones while non-agglutinated cells pass through during washing. Importantly, the pore diameter of each grade was essentially unchanged by the surface treatment. For both APTES and GPTMS, and across the full range of concentrations and reaction times, the pore size of the silanised papers matched that of the untreated papers to within the measurement scatter (Figure S5). This is expected, because the grafted silane forms only a thin, nanometre-scale molecular layer on the fibre surfaces, consistent with the modest silicon loadings reported above which is negligible relative to the micron-scale pores; the modification therefore preserves the native pore geometry and, with it, the size-exclusion filtration on which the trap-and-wash readout depends. Whatman No. 4 was therefore adopted for all device work. Beyond the contact-angle, SEM-EDS and FT-IR evidence presented here, covalent grafting through epoxide ring-opening is well established for GPTMS films by surface-sensitive X-ray photoelectron spectroscopy [23], and one-step, high-density covalent immobilisation of proteins and antibodies on epoxysilane-modified surfaces via the same epoxide ring-opening is well documented [22]. The specific antibody-cell capture is further demonstrated functionally in this work (Figure S7) and morphologically by SEM (Figure S8).

### 3.2. The APTES–Glutaraldehyde Chromophore

**Figure 4:**
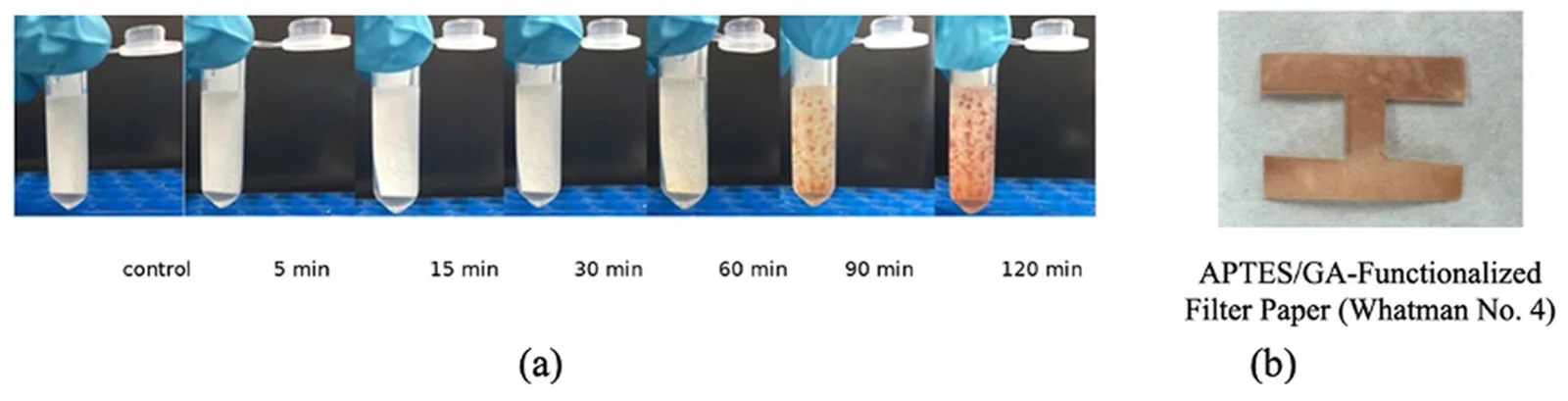
Time-dependent glutaraldehyde (GA) treatment of APTES-functionalised Whatman No. 4 filter paper. (**a**) Effect of GA treatment time (control and 5–120 min), with non-functionalised filter paper as the control; the red-brick colour deepens with time and is strong by 120 min. (**b**) Representative APTES/GA-functionalised filter paper (Whatman No. 4) after 120 min of GA treatment.

In the conventional APTES route, the surface amines must first be activated with glutaraldehyde before antibody coupling. When APTES-functionalised Whatman No. 4 was treated with 2.5% (*v*/*v*) glutaraldehyde (GA) and subsequently rinsed thoroughly with 100 mM phosphate-buffered saline (PBS, pH 7.4) to remove unreacted, free glutaraldehyde, the paper retained a pronounced red-brick colouration. A time-course of the GA treatment (Figure 4a; control and 5, 15, 30, 60, 90 and 120 min) showed little change at short times and a progressively deepening red-brick colour that became clearly visible by about 90 min and strong and uniform by 120 min; a representative APTES/GA-functionalised paper after 120 min is shown in Figure 4b. The fact that the colour survived this washing step and subsequent drying is significant. It establishes that the chromophore is retained on the cellulose itself, rather than arising from residual glutaraldehyde solution held within the pores. The effect was reproducible and was produced by neither the silanised paper nor the glutaraldehyde solution alone (the non-functionalised paper serving as the control), indicating that it originates specifically from the reaction between the surface amines and the aldehyde.

Because the resulting colour occupies the same red-orange region of the spectrum as trapped red blood cells, it masks the ABO colorimetric signal and renders both visual and red-channel readout unreliable. The APTES-GA-antibody pathway is therefore unsuitable for the intended device, irrespective of how reproducible the antibody coupling itself might be. The underlying chemistry is well established. Glutaraldehyde exists as an equilibrium of monomeric and oligomeric forms, and its reaction with surface amines through Schiff-base and Michael-type additions generates cross-bridged, conjugated products that absorb visible light—the same mechanism responsible for brown colour development in glutaraldehyde protein staining [24]. Importantly, this represents a context-dependent failure mode rather than a generic limitation of the chemistry. In 2025, a paper-based SARS-CoV-2 antibody assay deliberately exploited this same APTES-GA brown colouration as its analytical signal, the colour intensity correlating with antibody concentration [25]. The present findings and that report agree on the chemistry but arrive at opposite engineering conclusions. The chromophore is advantageous when the colour itself constitutes the readout, but is a failure mode when the readout depends on the red-channel transparency of the substrate, as in colorimetric ABO typing. We therefore highlight this incompatibility for future paper-based platforms operating in the same red-spectral window. An alternative immobilisation chemistry is required that is simultaneously covalent, activity-preserving and optically silent requirements satisfied by the GPTMS route examined below.

### 3.3. GPTMS Immobilisation, Condition Selection and Wash Optimisation

GPTMS presents a terminal epoxide group that reacts directly with the primary amines of the antibody (lysine ε-amines and the N-terminal α-amine) in a single mild step, forming a covalent C–N bond without any crosslinker, activator or coloured intermediate. Because an antibody carries numerous surface amines, coupling through one or a few sites remote from the antigen-binding region leaves recognition activity essentially intact, while the substrate itself remains optically clean. This route thus satisfies the covalent, activity-preserving and optically silent requirements identified in Section 3.2. The functional confirmation is provided below.

**Figure 5:**
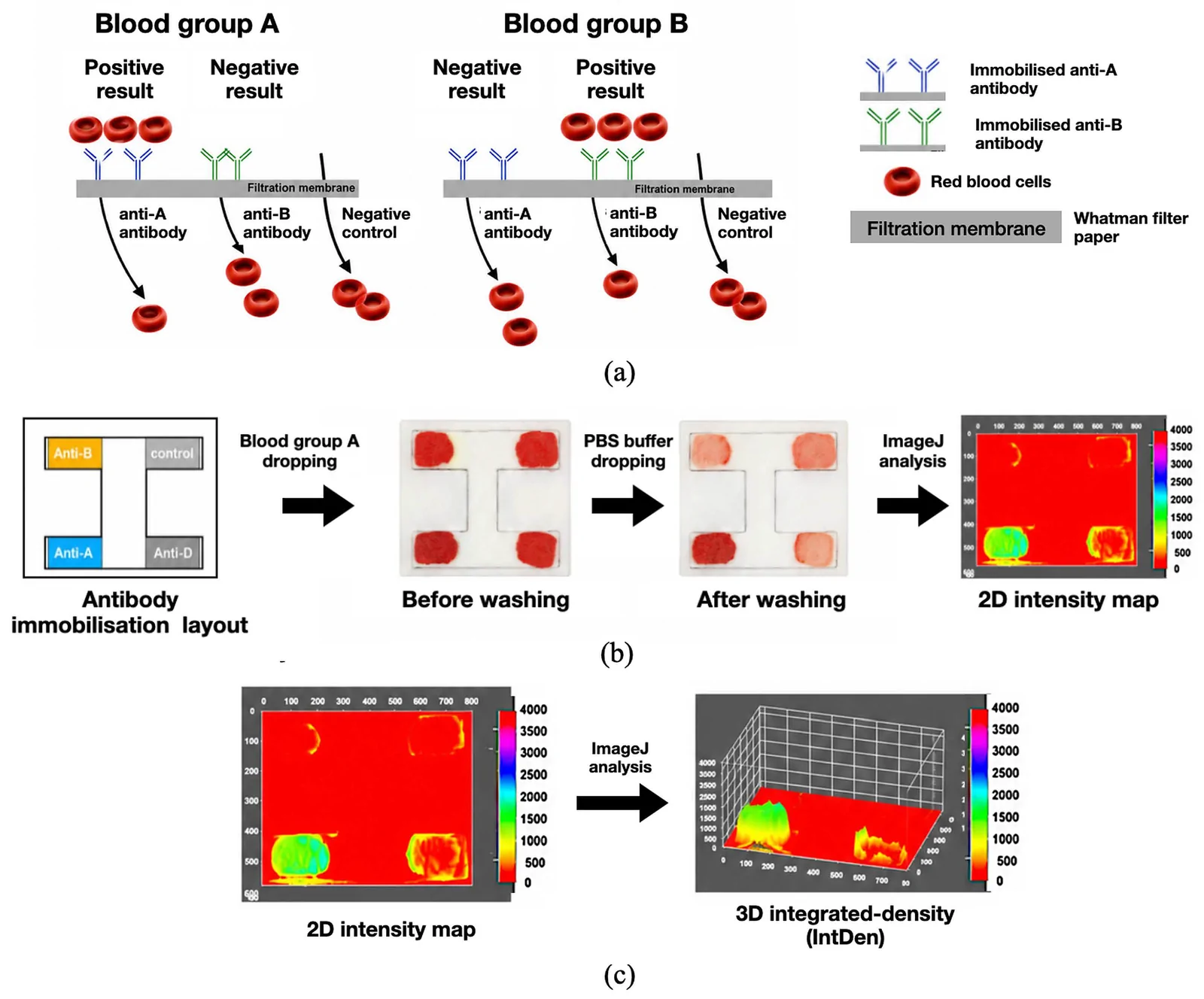
Principle and readout of the four-zone GPTMS assay (Whatman No. 4, 10% *v*/*v*, 3 h). (**a**) Trap-and-wash mechanism for blood groups A and B. Agglutinated red blood cells are retained on matched antibody zones (positive), while non-agglutinated cells pass through the membrane (negative). (**b**) Representative device before and after PBS washing. (**c**) ImageJ (v1.54)-based quantitative analysis using red-channel intensity and 3D integrated-density (IntDen) mapping of Blood group A.

**Figure 6:**
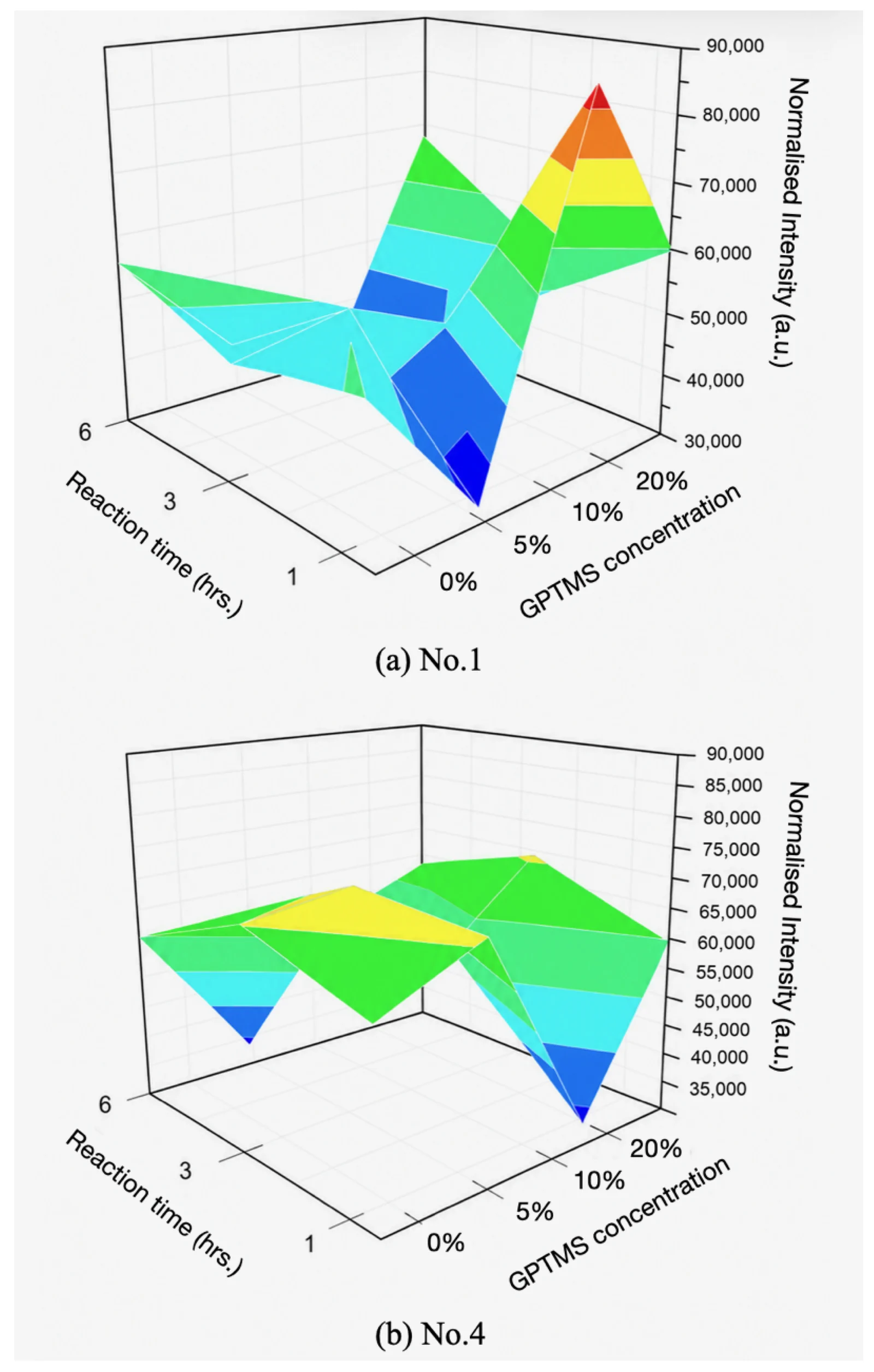
Normalised red-channel intensity of the positive zone (blood group A on anti-A) after PBS washing, mapped using ImageJ (v1.54) against GPTMS concentration and reaction time for Whatman filter paper (**a**) No. 1 and (**b**) No. 4. Blue–purple, cyan–green, and yellow–red indicate low, intermediate, and high intensity, respectively.

The working GPTMS condition was selected on the basis of the ABO readout itself, which traps agglutinated cells on the matched antibody zones while non-agglutinated cells wash through the paper (Figure 5a,b). Papers of the two grades (pore sizes 11 and 20 µm) were functionalised across the full concentration (5%, 10% and 20% *v*/*v*) and time (1, 3 and 6 h) grid and tested; the red-channel integrated density (IntDen) was extracted from each zone by ImageJ (v1.54) processing of the washed device (Figure 5c). The normalised intensity of the positive (Blood group-A/anti-A) zone was mapped as a three-dimensional surface against GPTMS concentration and reaction time for each paper grade (Figure 6), and the positive-minus-negative difference was evaluated from the matched positive and negative zones. The largest single difference was obtained for the 11 µm paper at GPTMS 20% *v*/*v* and 1 h, followed closely by the 20 µm paper at 10% *v*/*v* and 3 h. A larger difference corresponds to a clearer and more robust readout.

Pore geometry and wash-through behaviour then discriminated between the two leading conditions. Although the 11 µm paper afforded a marginally larger raw contrast, its pores are close to the red-cell diameter and drain the PBS wash slowly, whereas the 20 µm paper (Whatman No. 4) drains considerably faster. For six PBS washes the 20 µm paper cleared in 106 s in total, compared with 805 s for the 11 µm paper. Because faster and more complete wash-through yields sharper negative zones and a more practical device, GPTMS at 10% *v*/*v* and 3 h on Whatman No. 4 was adopted as the working condition.

The wash step that underlies this contrast also has a clear mechanical basis, which informed its optimisation. Whether a zone reads positive or negative depends on whether red cells remain attached to the antibody-bearing surface once the PBS wash is applied. The antigen–antibody interaction is a specific lock-and-key bond, but its mechanical strength is modest. The bond between a single antigen on a red cell and a surface-bound antibody has been measured at only about 20–30 pN in classic red-cell tube-flow experiments [26], and both molecular-point attachments and larger agglutinin-bonded contacts between red cells are ruptured once a comparable mechanical force is applied [27,28]. A red blood cell is comparatively large (≈7–8 µm in diameter), so as the buffer front passes it experiences a substantial viscous drag and surface-tension force; for a non-agglutinated cell held by only one or a few antibody bridges, this force readily exceeds the available bond strength and pulls the cell away from the surface, clearing the mismatched zone, since a single lock-and-key contact cannot anchor a micron-scale cell against the wash. Agglutinated cells behave differently. IgM antibodies cross-link them into a multivalent three-dimensional network, so the mechanical load is shared over many bonds, and the aggregate is simultaneously trapped within the pores of the larger-grade paper and retained. A wash that is strong enough to strip individual cells yet not so aggressive as to dislodge the agglutinated network therefore maximises the positive-to-negative contrast, which is why the fast-draining, larger-pore Whatman No. 4 gave the most robust readout.

In operational terms, the selected six-cycle 100 µL PBS protocol consumes 600 µL of buffer per zone and completes the wash in about 106 s, compared with roughly 805 s for the 20-cycle 50 µL alternative that gave poorer contrast. The four zones are washed simultaneously rather than sequentially. The buffer applied to each zone permeates vertically through the filter paper into the underlying absorbent layer and carries the non-agglutinated cells away together, giving a total buffer consumption of about 2.4 mL per device. The procedure requires only a few manual steps applying the RBC sample to the four zones, performing the simultaneous wash and imaging the device, with no pipetting on an open strip, and the complete assay is performed within a few minutes.

**Figure 7:**
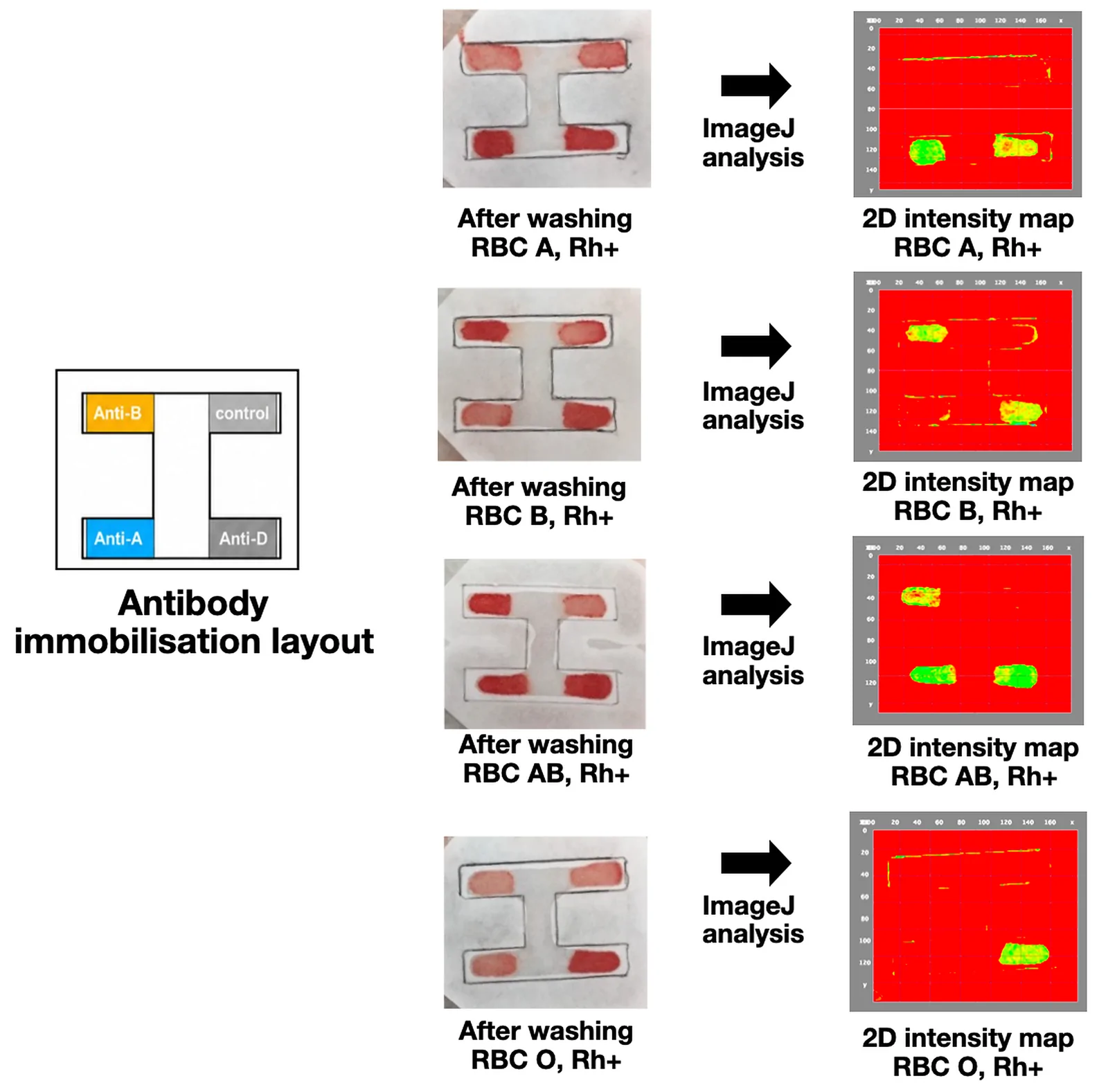
Antibody-immobilisation layout and ImageJ (v1.54) false-colour intensity maps of RhD-positive A, B, AB, and O red blood cells. In the maps, red indicates low intensity, whereas yellow and green indicate progressively higher red-channel intensity. Group-specific retention at the corresponding antibody zones enables ABO typing.

The contrast was governed by the wash volume and not solely by the number of cycles. With 50 µL washes over 20 cycles, the control and negative IntDen values failed to stabilise, so that no reliable endpoint could be defined. With 100 µL washes, the control and negative signals reached a stable baseline from the sixth wash onward and remained there, defining a six-cycle 100 µL protocol. Volumes exceeding 100 µL per cycle were counter-productive: the excess PBS disturbed antigen–antibody binding and drove even reacted cells through the pores, degrading the readout. The 6 × 100 µL protocol was therefore used throughout the subsequent work. Under the optimised conditions, the specificity of the immobilised antibodies was evaluated using RhD-positive reagent red blood cells from each ABO group (Figure 7). After sample application and washing, ImageJ (v1.54) analysis revealed antigen-specific retention: group A cells were retained at the anti-A and anti-D zones; group B at anti-B and anti-D; group AB at anti-A, anti-B and anti-D; and group O at anti-D only. The antibody-free control remained negative for all samples. These distinct retention patterns confirm that GPTMS-immobilised antibodies maintain their binding specificity on the paper substrate, enabling selective ABO typing and supporting the subsequent quantitative and clinical evaluations.

**Figure 8:**
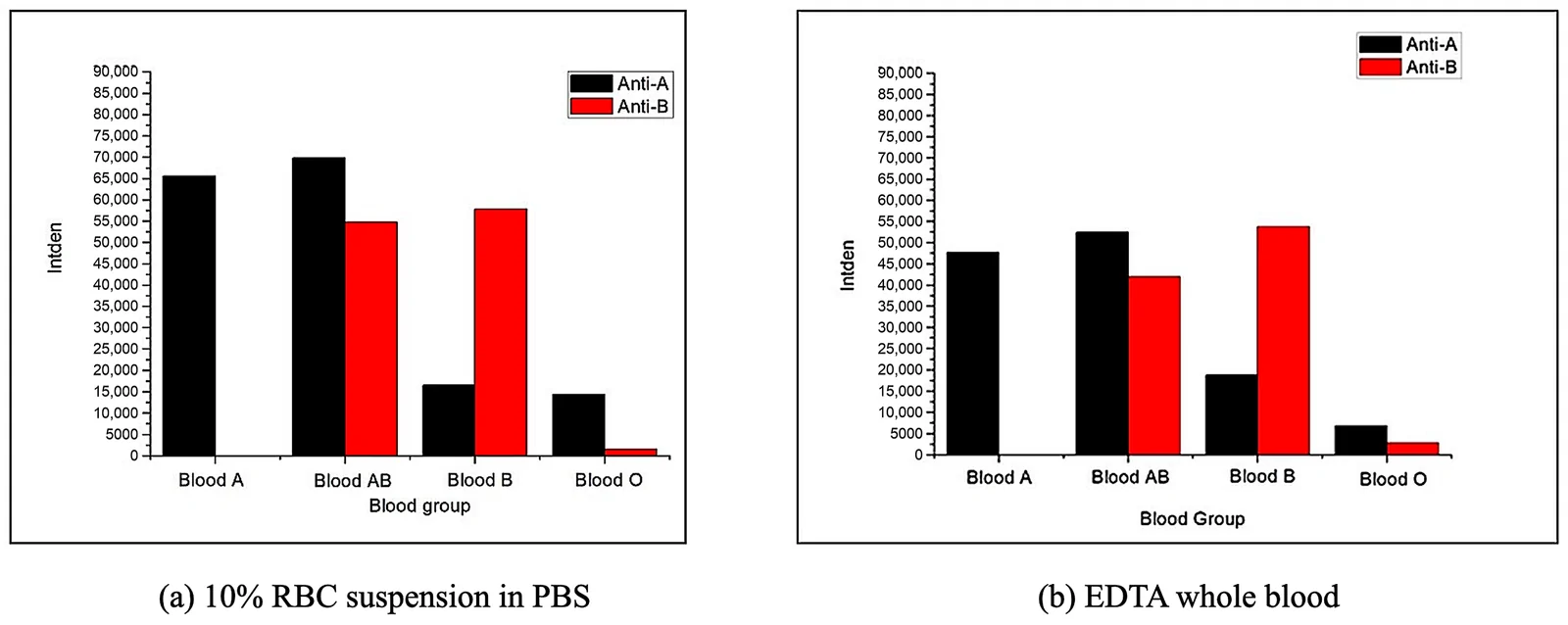
Red-channel IntDen across the four ABO groups for (**a**) a 10% RBC suspension in PBS and (**b**) EDTA whole blood; the two sample formats give equivalent diagnostic patterns (the absolute intensity being somewhat lower for whole blood), confirming that the assay works directly on anticoagulated clinical blood.

To verify that the device functions with anticoagulated clinical blood, and not only with reconstituted reagent cells, RBC-and-plasma samples and EDTA whole blood of all four groups were compared. Both sample types produced the same diagnostic pattern the correct zones positive and the others at baseline so that every group was typed correctly from either format, as shown in Figure 8. The functionalised paper therefore reads both reconstituted and EDTA-anticoagulated blood, the latter being the format routinely encountered in practice. The absolute red-channel intensity, however, was consistently somewhat lower for EDTA whole blood than for the 10% RBC suspension in PBS. This reduction is attributable to the complex protein matrix of whole blood. Plasma proteins such as albumin, immunoglobulins and fibrinogen adsorb non-specifically onto the cellulose and onto the antibody layer and partially block the antibody sites, so that fewer red cells are captured and retained at each zone. Thus, the measured colour scales with the number of retained cells; this lowers the red-channel IntDen relative to the protein-free PBS suspension. Such matrix fouling is a well-recognised cause of reduced capture and signal in antibody-based assays performed directly in plasma or whole blood [9,29]. Crucially, the effect reduced the magnitude of the signal without altering the pattern: because GPTMS anchors the antibody covalently and stably to the cellulose rather than by physisorption, which is readily displaced by plasma proteins, the recognition remained specific and the correct zones stayed clearly positive above the control baseline. The controlled surface chemistry is therefore the key determinant of a clean, matrix-tolerant readout [19], and it is what allows the device to type EDTA whole blood correctly despite the lower absolute intensity.

A practical limitation was identified and is reported explicitly. Antibody-functionalised inserts remained usable for up to seven days under refrigeration. After this period, the immobilised antibody dried on the paper and the readout became faint and unreliable, even though a difference between the pre- and post-wash colours was still apparent. The inserts are therefore best used immediately after antibody spotting. Sealing the functionalised paper within a moisture-, light- and air-barrier aluminium pouch is proposed as a means of preserving activity, and the development of an ambient-stable reagent formulation is identified as necessary future work prior to field deployment. This finding qualifies any claim of a ready-to-store consumable and is important for a realistic assessment of shelf life.

### 3.4. Device Design and Operation

**Figure 9:**
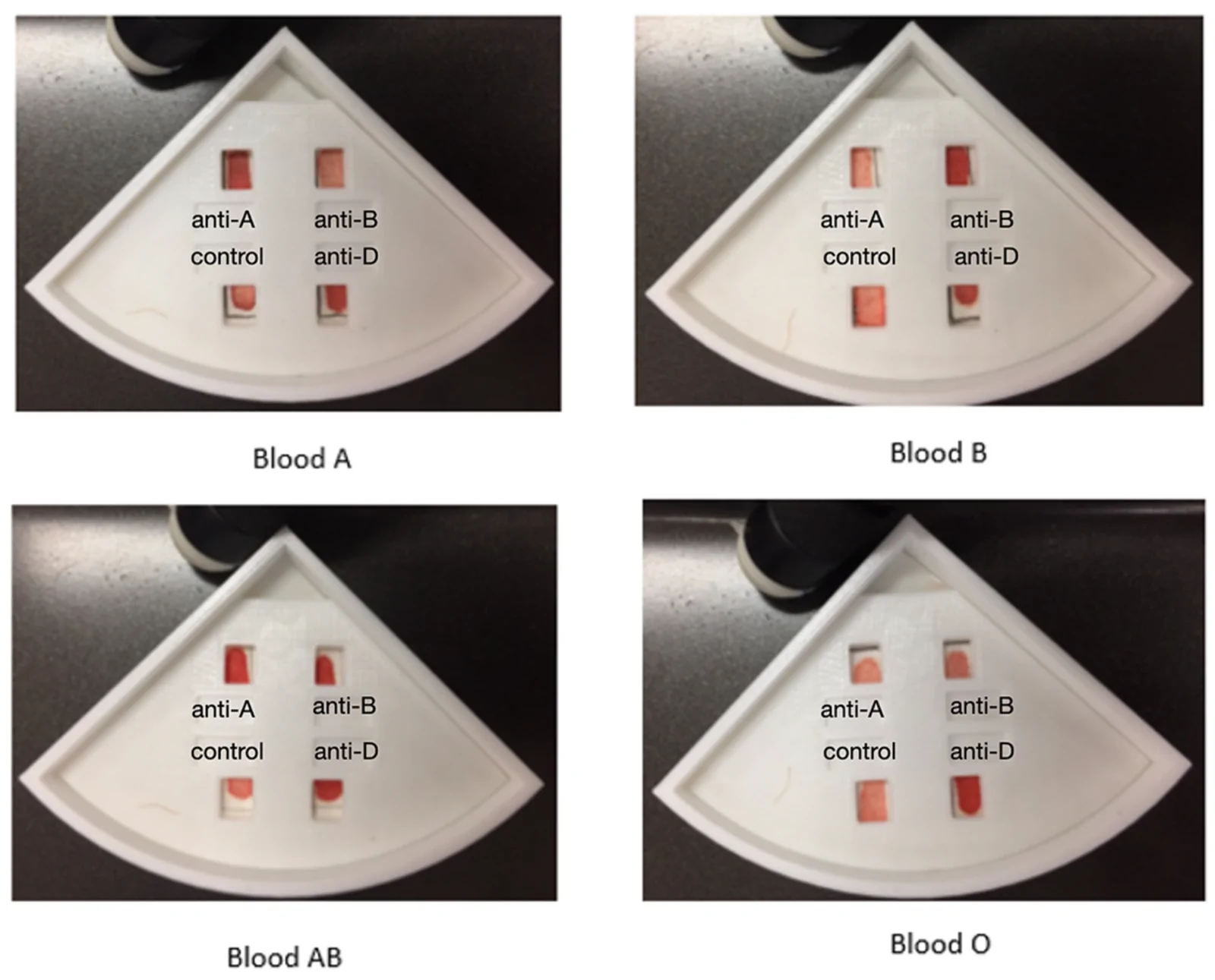
The assembled 3D-printed device tested with the four ABO groups (blood A, B, AB and O), showing the expected agglutination pattern in each case.

The functionalised insert was integrated into a self-contained, 3D-printed housing so that the assay could be performed without pipetting on an open strip. Each insert is cut as one quadrant of a circular Whatman disc, so that a single disc yields four inserts with no waste; its four zones anti-A, anti-B, anti-D and an antibody-free control are defined by an H-shaped silicone barrier applied prior to antibody spotting (Figure S6). The fan-shaped housing comprises two stacked compartments. The upper compartment seats the insert and exposes the four zones through windows in the top face, while the lower compartment holds a two-layer stack of untreated absorbent paper. When PBS is dispensed onto each zone, gravity and capillary action draw the buffer and non-agglutinated cells downward into the absorbent layer, whereas agglutinated cells are retained on the zones above. This arrangement converts the wash from a lateral-flow process into a vertical one and keeps the imaged surface free of standing liquid, so that the sharp positive/negative contrast established in Section 3.3 can be read directly from the device (Figure 9). The fixed window positions further allow a single region-of-interest template to be applied across all images—a consideration relevant to the illumination sensitivity discussed in Section 3.5. Full fabrication details are provided in the Supplementary Information.

### 3.5. Validation on 80 Blood Samples

**Figure 10:**
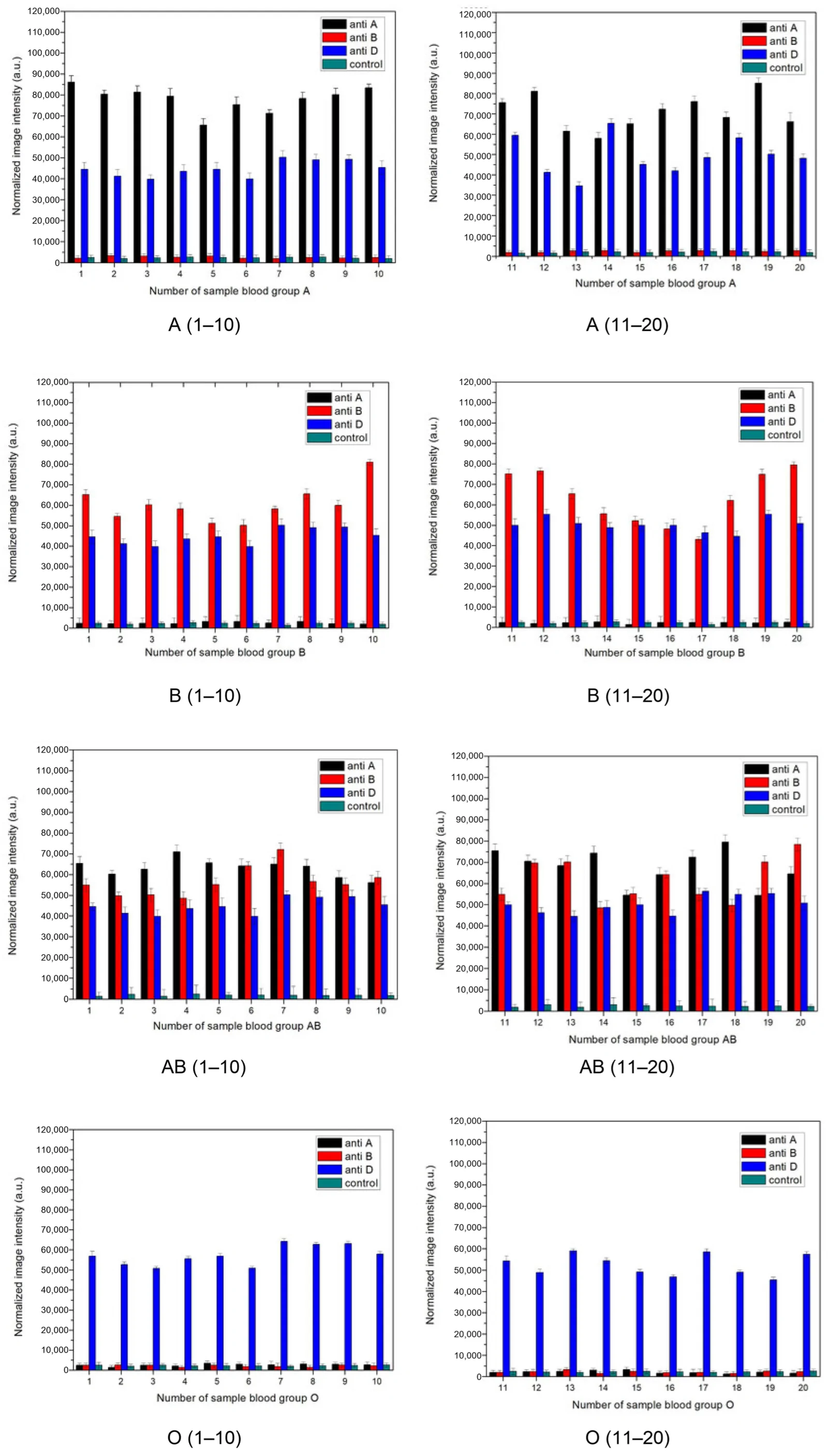
Quantitative ABO-RhD validation on the 80 clinical samples. Normalised red-channel image intensity (a.u., ImageJ (v1.54)) of the anti-A, anti-B, anti-D and control zones for the 20 samples of each ABO group (samples 1–10 and 11–20 shown separately): group A (anti-A and anti-D positive), group B (anti-B and anti-D positive), group AB (anti-A, anti-B and anti-D positive) and group O (anti-D positive only). In every sample the diagnostic zones are strongly positive while the remaining zones stay at the antibody-free control baseline, giving 80/80 correct assignments.

The complete device comprising the GPTMS-functionalised insert (10% *v*/*v*, 3 h; Whatman No. 4), the four-zone antibody layout, and the 6 × 100 µL PBS washing protocol was evaluated using 80 EDTA-anticoagulated blood samples obtained from Chonburi Hospital, with 20 samples each from blood groups A, B, AB, and O. For each sample, the reaction pattern across the anti-A, anti-B, and anti-D zones was quantified using red-channel integrated density (IntDen). The resulting reaction profile was used to assign the predicted ABO blood group, which was subsequently compared with the corresponding reference blood-group result. The four ABO groups exhibited their expected antigen–antibody reaction patterns, while the anti-D zone was positive for all samples, consistent with all 80 specimens being classified as RhD-positive (Figure 10). The device correctly classified all 80 samples, with 20/20 correctly identified samples for each of groups A, B, AB, and O (Table 1), corresponding to an overall classification accuracy of 100% (95% CI: 95.4–100%, Wilson method). The ImageJ (v1.54)-based classifications were in complete agreement with the unaided visual interpretations.

Consistent with this complete agreement between the device predictions and the reference classifications, the 4 × 4 confusion matrix contained observations exclusively along the main diagonal. For each ABO group, sensitivity, specificity, positive predictive value (PPV), and negative predictive value (NPV) were all 100%. Sensitivity was 20/20 (95% CI: 83.9–100%), while specificity was 60/60 (95% CI: 94.0–100%), with confidence intervals calculated using the Wilson method. In addition to categorical blood-group classification, the net integrated density (net-IntDen) measured at each reaction zone provided a quantitative measure of the corresponding ABO or anti-D reaction (Figure 10). Across the evaluated cohort, all diagnostic ABO and anti-D reactions exhibited consistently high net-IntDen values, supporting the qualitative classification results.

The colorimetric readout is a direct physical consequence of the agglutination reaction, and it is worth making this link explicit. In forward ABO typing, IgM antibodies directed against a red-cell surface antigen cross-link the cells into large aggregates. The extent of this reaction, the agglutination strength, conventionally graded from strong to weak, governs how efficiently the cells are retained when the buffer washes through. Agglutinated cells are fixed on and within the antibody-bearing paper, so the local red colour, and hence the red-channel integrated density, scales with the number of red cells trapped at the zone, whereas non-agglutinated cells are eluted and leave the zone pale [8,9]. A positive result is thus reported as a high, retained red intensity and a negative result as a near-baseline intensity, and the magnitude of the positive signal reflects the underlying agglutination strength. This coupling between agglutination strength, red-cell retention on the cellulose and the measured colour intensity is what allowed the four-zone pattern of each sample to be assigned unambiguously and quantitatively, and it underlies the 100% concordance obtained across the 80 samples.

Positive and negative zone calls were determined by background subtraction against the control-channel signal: the background-corrected classification and the unaided visual read agreed for all 80 samples, with every zone falling clearly on the positive or negative side of the threshold. The constant illumination and fixed imaging geometry maintained throughout were important in this respect, since the quantitative red-channel read is inherently sensitive to lighting; the device’s fixed windows and single region-of-interest template (Section 3.4) make this control straightforward to sustain. The result is nonetheless reported with appropriate caution. With 20 samples per group, the exact 95% confidence intervals remain wide (100% and 83.9–100%, Wilson), every sample originated from a single hospital, and all samples were RhD-positive, so the anti-D channel is validated here for the positive call only. A larger, multi-site cohort that includes RhD-negative and weak-D samples and ABO subgroups (for example, A1/A2) will be required before this perfect concordance can be generalised. RhD-negative and weak-D phenotypes are, moreover, uncommon in the Thai population and generally require molecular methods for confirmation [30], which is why the present serological cohort is RhD-positive; indeed, no RhD-negative specimen was present among the approximately 200 samples available.

Considered in the context of the literature, the contribution of the platform is twofold: a covalent, optically silent GPTMS immobilisation that avoids the glutaraldehyde chromophore failure mode documented in Section 3.2, and a self-contained two-compartment housing that manages the wash without external fluidics. The device typed all 80 hospital samples correctly by both eye and camera, matching or exceeding the accuracy reported for related paper-based ABO devices. The remaining limitations a single-source cohort, RhD validated for the positive call only, the absence of A subgroups and weak-D samples, and an insert shelf life of about one week define a clear path toward a deployable test.

These design choices can be placed in the context of several recent directions in paper-based diagnostics. In device architecture, vertically stacked paper microarrays have been used to raise throughput and multiplexing, for example for simultaneous SERS detection of two cancer biomarkers [31]; the present two-compartment housing pursues the same vertical organisation of flow, but to manage the wash and trap agglutinates rather than to multiplex labels. In readout, deep-learning-enhanced paper vertical-flow assays with nanoparticle amplification have achieved high-sensitivity quantitative detection of cardiac troponin [32]; the quantitative element here is deliberately lighter—a fixed-camera red-channel read with control-zone background subtraction so that the assay stays instrument-light while still giving an objective, uniform signal. In reagent stability, a paper fluorescent assay using long-term ambient-stored bioengineered red blood cells has recently been reported for blood typing and antibody-titre determination [33]; this is directly relevant to the one-week shelf-life limitation of the antibody-loaded insert noted above and points to a concrete route toward an ambient-stable version of the present device. Positioned against these advances, the contribution of this work is a covalent, optically silent surface chemistry combined with a trap-and-wash vertical housing that delivers signal uniformity without added labels or instrumentation.

A first-order cost estimate reinforces the low-resource positioning of the platform. At laboratory scale, the direct consumable cost is approximately 0.33 USD per test, based on current bench purchase prices and measured per-test usage, and is dominated by the cellulose substrate and the disposable 3D-printed insert, with the antibody aliquots, wash buffer and other reagents each contributing a smaller share (the outer housing is reusable). These are laboratory-scale figures and should be read as an upper bound: at manufacturing scale, bulk reagent procurement, injection-moulded housings in place of 3D printing and automated dispensing would be expected to lower the per-test cost further through economies of scale.

## 4. Conclusions

An integrated, instrument-light paper-based device for ABO blood typing has been developed and, in doing so, this work documents a surface-chemistry pitfall of relevance to the wider field. In comparing APTES and GPTMS on Whatman cellulose, APTES was found to form a strongly hydrophobic layer that impedes aqueous wicking, and its conventional glutaraldehyde activation to generate a red-brick chromophore on cellulose—a context-dependent failure mode for colorimetric blood typing, given that the same chromophore serves elsewhere as an analytical signal. GPTMS avoids this problem entirely, coupling antibodies in a single mild step without a crosslinker and without a visible colour. Guided by the positive-versus-negative signal contrast and by the PBS wash-through time, GPTMS (10% *v*/*v*, 3 h, Whatman No. 4) was selected in combination with a six-cycle 100 µL PBS wash, and the functionalised insert was integrated into a 3D-printed two-compartment housing that manages the wash vertically. On 80 hospital samples the device classified every sample correctly (100%; 95% CI 95.4–100%), the ImageJ (v1.54) and unaided visual reads agreeing throughout under controlled illumination. Mechanistically, the platform works because the covalent GPTMS layer keeps the antibody functional within the whole-blood matrix, while the gentle wash exploits the weakness of an individual antigen–antibody bond to strip non-agglutinated cells, which a single lock-and-key contact cannot hold against the hydrodynamic force on a micron-scale cell, yet retains the multivalently cross-linked agglutinates, so that the retained red intensity reports the agglutination strength directly. The principal limitations are the single-source cohort (80 samples from one hospital); RhD confirmed for the positive call only, with no weak-D or ABO subgroups tested; and an insert shelf life of about one week under refrigeration. Extending validation to a larger, multi-site cohort that includes RhD-negative and weak-D samples and ABO subgroups, and formulating an ambient-stable, pouch-sealed insert, are the natural next steps toward a field-deployable test. Even at the present stage, the covalent, optically clean GPTMS chemistry and the self-contained device together provide a low-cost, visually readable route to ABO typing for point-of-care and resource-limited settings.

## Figures and Tables

**Table 1:** Diagnostic performance of the device on 80 clinical blood samples (20 per group) against the reference typing result. The 95% confidence intervals (CIs) are Wilson intervals. All samples were RhD-positive, so the anti-D channel is validated for the positive call only.

Blood Group	Samples	Correct	Accuracy (%)	95% CI
A	20	20	100	83.9–100
B	20	20	100	83.9–100
AB	20	20	100	83.9–100
O	20	20	100	83.9–100
**Overall**	**80**	**80**	**100**	**95.4–100**

## Data Availability

The original contributions presented in this study are included in the article and Supplementary Material. The raw ImageJ (v1.54) integrated-density measurements, SEM-EDS spectra and device images supporting the reported results are available from the corresponding author upon reasonable request. The clinical blood samples cannot be shared, as they were provided under an ethical approval that restricts their use to this study.

## Supplementary Materials

The following supporting information can be downloaded at: https://www.mdpi.com/article/10.3390/bios16090468/s1, Figure S1: water contact-angle images of APTES-functionalised paper; Figure S2: time-dependent water contact angles of GPTMS-functionalised paper; Figure S3: SEM micrographs and SEM–EDS spectra of APTES-functionalised paper; Figure S4: SEM micrographs and SEM–EDS spectra of GPTMS-functionalised paper; Figure S5: pore-diameter analysis of untreated and silanised Whatman No. 1 and No. 4; Figure S6: design of the 3D-printed two-compartment device; Figure S7: comparison of type-A RBC retention on GPTMS-functionalised, non-functionalised, APTES-functionalised and GA-treated Whatman No. 4 filter paper after washing, with ImageJ (v1.54) analysis, confirming specific antibody immobilisation only on the GPTMS route. Figure S8: SEM images of GPTMS-functionalised paper showing type-A RBC interaction with immobilised antibodies (positive in the anti-A zone, negative in the anti-B zone).
