# Supplementary material for: An Optically Silent Epoxysilane Chemistry for Paper-Based ABO Blood Typing
Source: Biosensors (Basel). 2026 Aug 27;16(9):468. doi: 10.3390/bios16090468 (PMC13604687; doi:10.3390/bios16090468)
Supplement: Supplementary file 1 [file biosensors-16-00468-s001.zip › biosensors-4488073-supplementary.pdf]

# Supplementary Information

*An optically silent epoxysilane chemistry for paper-based ABO blood typing*

Chinnawut Pipatpanukul[1*], Komkrisd Wongtimnoi[1], Laurent Mezeix[1], Santi Phosri[2]

[1]*Department of Advanced Materials Engineering, Faculty of Engineering, Burapha University, Chonburi, Thailand*

[2]*Department of Chemical Engineering, Burapha University, Chonburi, Thailand*

*Corresponding author: chinnawut.pi@buu.ac.th

## Section S1. Surface characterisation: contact angle, SEM–EDS and pore size

Water contact angle (WCA) was used to follow the change in surface wettability on silanisation. On APTES-functionalised paper the droplet beaded up and the contact angle remained close to 120° for at least 30 min, confirming a stable, hydrophobic aminosilane layer (Figure S1). On GPTMS-functionalised paper the droplet was absorbed almost immediately, so that no stable angle could be measured; the epoxysilane layer therefore preserves the wicking of the untreated cellulose (main text, Section 3.1).

Scanning electron microscopy with energy-dispersive X-ray spectroscopy (SEM–EDS) was used to image the fibre morphology and to quantify the surface elemental composition after grafting. Both silanes increased the surface silicon content relative to the untreated paper. The APTES spectra additionally show a nitrogen signal arising from the aminopropyl group (Figure S2), which is absent from the GPTMS spectra, consistent with the epoxysilane structure (Figure S3).

The apparent pore diameter of the two paper grades was measured before and after silanisation to guide the paper-grade selection: Whatman No. 1 (≈ 11 µm) has pores only marginally larger than the ~7.5 µm red-cell diameter and tends to retain non-agglutinated cells, whereas the larger pores of No. 4 (≈ 20 µm) allow free cells to wash through (Figure S4). For both grades, the pore diameter was essentially unchanged by APTES or GPTMS modification across all concentrations and reaction times, confirming that the thin silane layer preserves the native pore geometry (main text, Section 3.1).

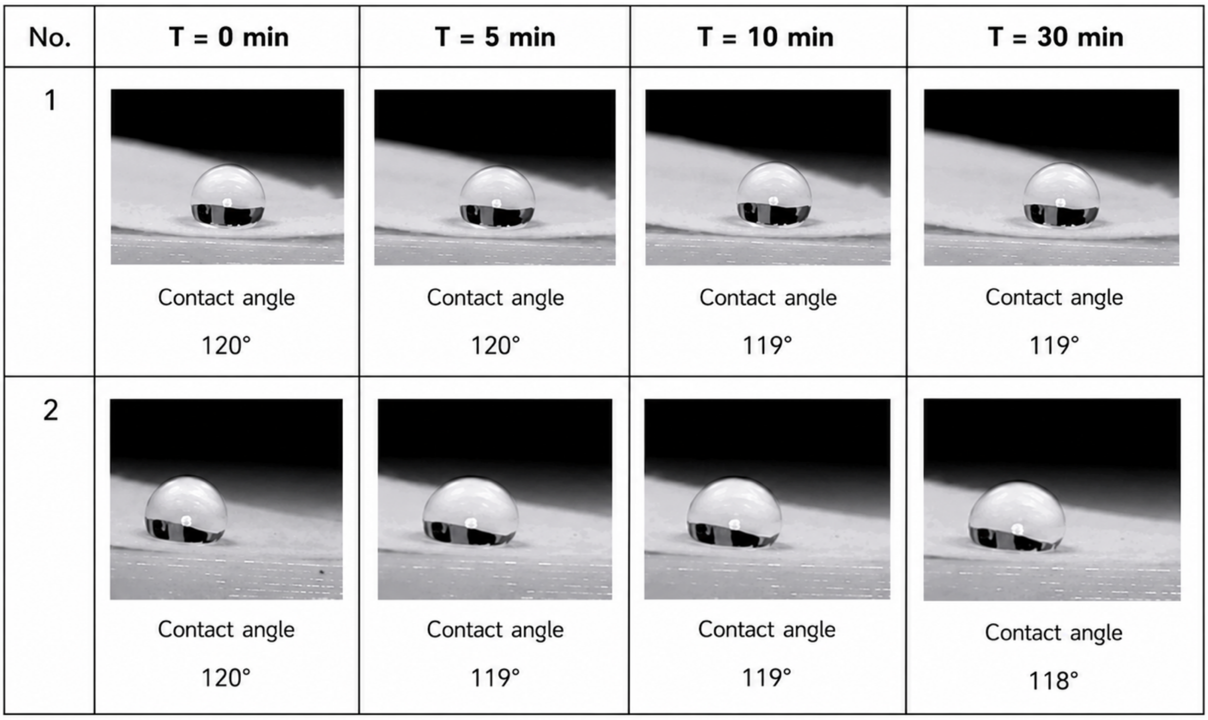


**Figure. S1.** Representative water contact-angle images of APTES-functionalised Whatman No. 1 and No. 4 (20% v/v, 1 h), recorded 0, 5, 10 and 30 min after droplet deposition. The measured angle remains close to 120° throughout, confirming that the APTES surface is hydrophobic and does not wick the droplet. Referenced in Section 3.1.1.

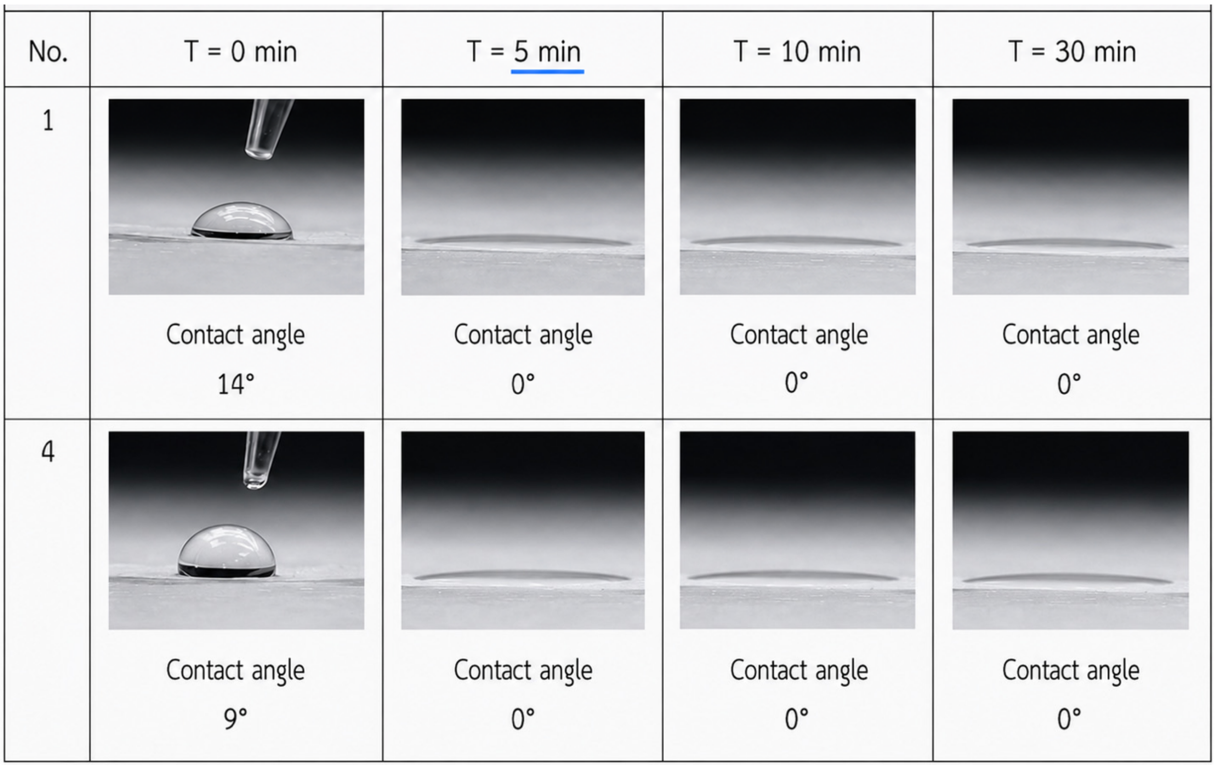


Figure. S2. Time-dependent water contact angles of GPTMS-functionalised Whatman filter papers (10% v/v, 1 h) after water deposition. Contact angles were recorded immediately (0 min) and after 5, 10 and 30 min for Whatman No. 1 and Whatman No. 4. The water droplets rapidly spread and reached complete wetting (0°) within 5 min for both filter papers.

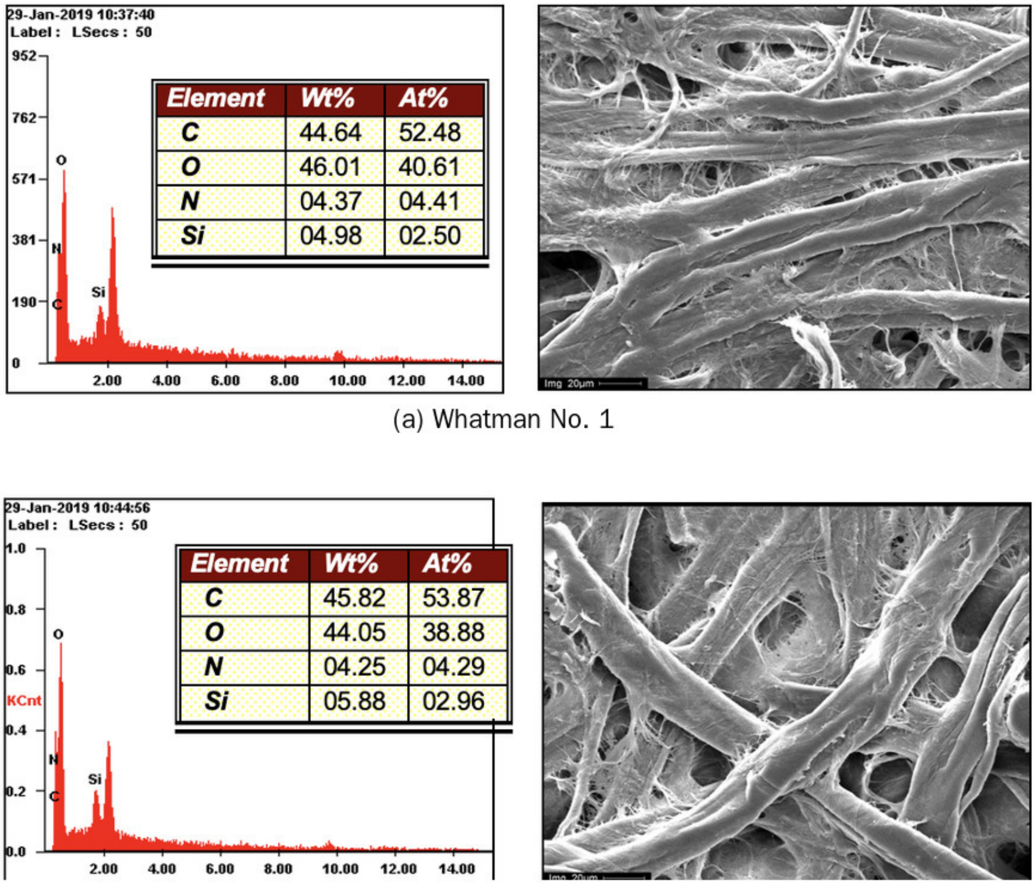


| Element | Wt% | At% |
|---|---|---|
| C | 44.64 | 52.48 |
| O | 46.01 | 40.61 |
| N | 04.37 | 04.41 |
| Si | 04.98 | 02.50 |

(a) Whatman No. 1

| Element | Wt% | At% |
|---|---|---|
| C | 45.82 | 53.87 |
| O | 44.05 | 38.88 |
| N | 04.25 | 04.29 |
| Si | 05.88 | 02.96 |

(b) Whatman No. 4

**Figure. S3.** SEM micrographs (500×, scale bar 20 µm) and the corresponding EDS spectra and elemental-composition tables for APTES-functionalised (a) Whatman No. 1 and (b) Whatman No. 4 (10% v/v, 3 h). The nitrogen (N) signal reflects the aminopropyl group of APTES. Referenced in Section 3.1.2.

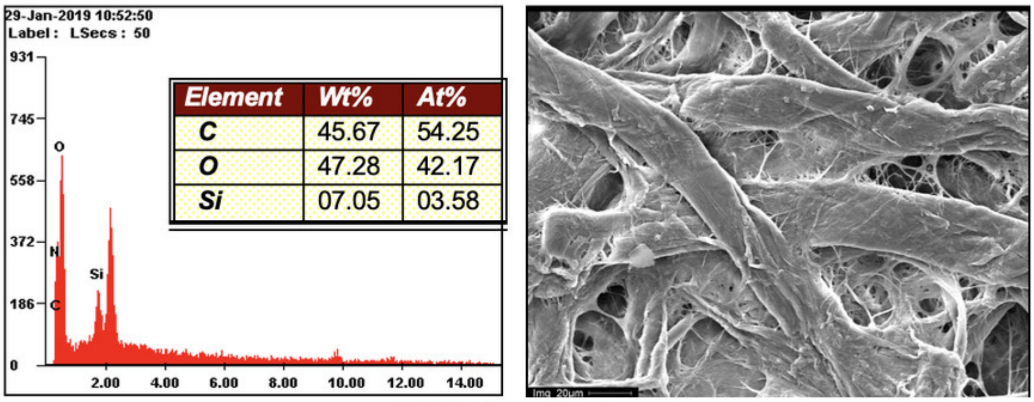


(a) Whatman No. 1

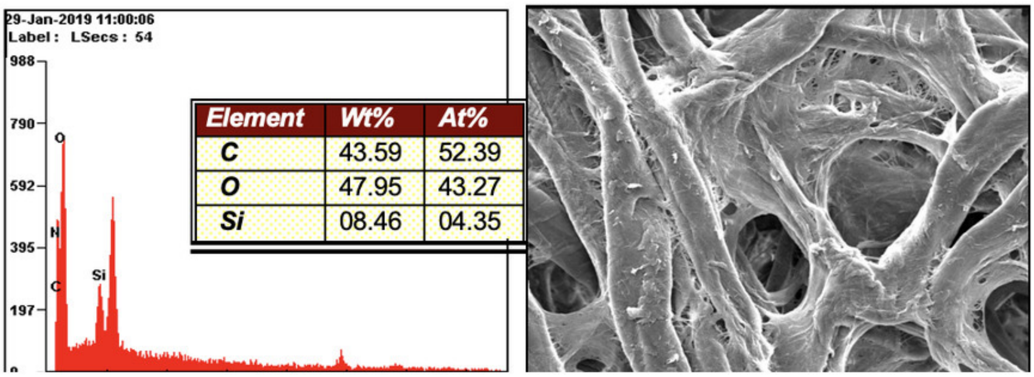


(b) Whatman No. 4

**Figure. S4.** SEM micrographs (500×, scale bar 20 µm) and the corresponding EDS spectra and elemental-composition tables for GPTMS-functionalised (a) Whatman No. 1 and (b) Whatman No. 4 (10% v/v, 3 h). No nitrogen signal is present, consistent with the epoxysilane. Referenced in Section 3.1.2.

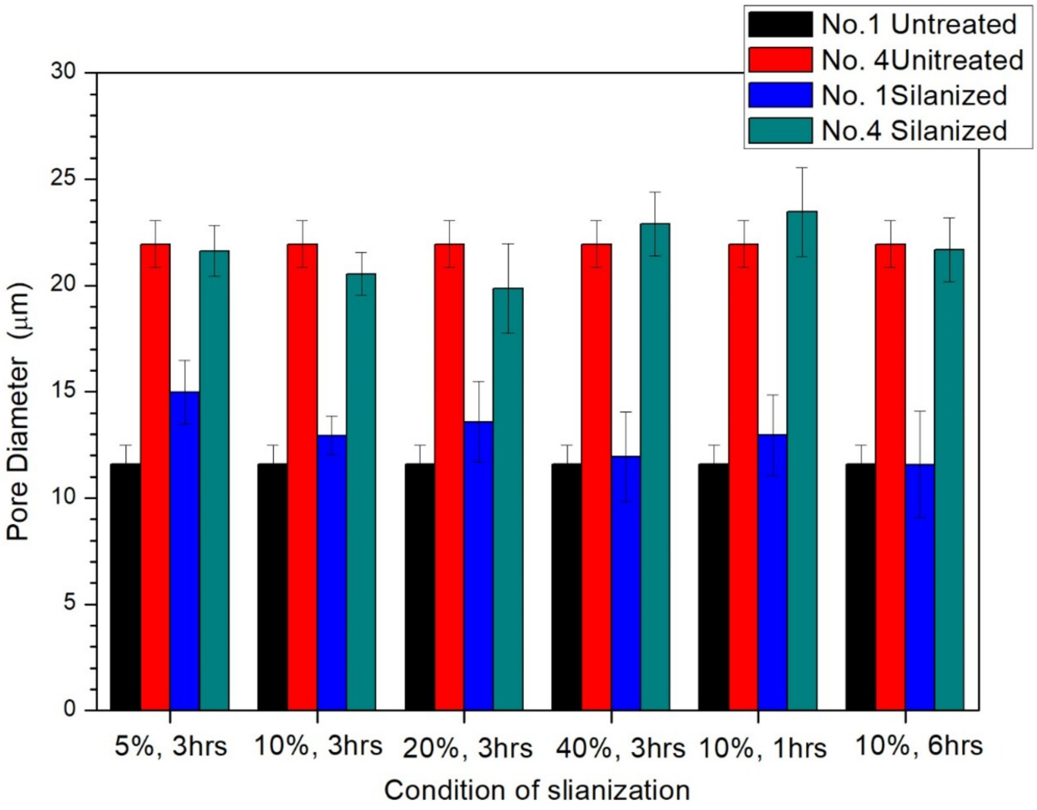


**Figure. S5.** Pore-diameter analysis of Whatman No. 1 and No. 4 papers, untreated and after silane functionalisation, across the range of APTES/GPTMS concentrations and reaction times. Silanisation does not appreciably change the pore diameter relative to the untreated papers (No. 1 ≈ 11 μm; No. 4 ≈ 20 μm), confirming that the modification preserves the pore geometry that governs red-cell filtration. Referenced in Section 3.1.2.

**Section S2. The 3D-printed device**

A portable two-compartment housing was designed to match the fan-shaped (approximately quarter-circle) geometry of the paper insert, which was cut so that a single circular Whatman disc yields four inserts. The upper compartment seats the antibody-functionalised insert and exposes its four zones (anti-A, anti-B, anti-D and control) through windows in the top face; the lower compartment holds a stack of untreated absorbent paper that wicks the PBS wash and eluted cells vertically away from the test surface, keeping the imaged surface free of standing liquid. The reusable housing was fabricated by fused-filament fabrication (FFF; also known as fused-deposition modelling, FDM) using poly(lactic acid) (PLA) filament of 1.75 mm diameter on a FlashForge Creator Pro (Zhejiang Flashforge 3D Technology, China).

Fused-filament fabrication with PLA was chosen because it is low-cost, widely accessible and adequate for a single-use insert together with a reusable outer housing; the fan-shaped layout also maximises material utilisation, since one circular Whatman disc yields four inserts. The design was kept free of support structures to simplify fabrication. Printing used a 0.4 mm nozzle, a layer height of 0.20 mm and a 20% grid infill, with nozzle and build-plate temperatures of 220 and 55 °C, respectively, and the standard slicing profile; the approximate print time was 30 min per housing, depending on component geometry and orientation. For production at scale, the same geometry could be injection-moulded, which would lower the per-part cost and cycle time relative to FFF printing.

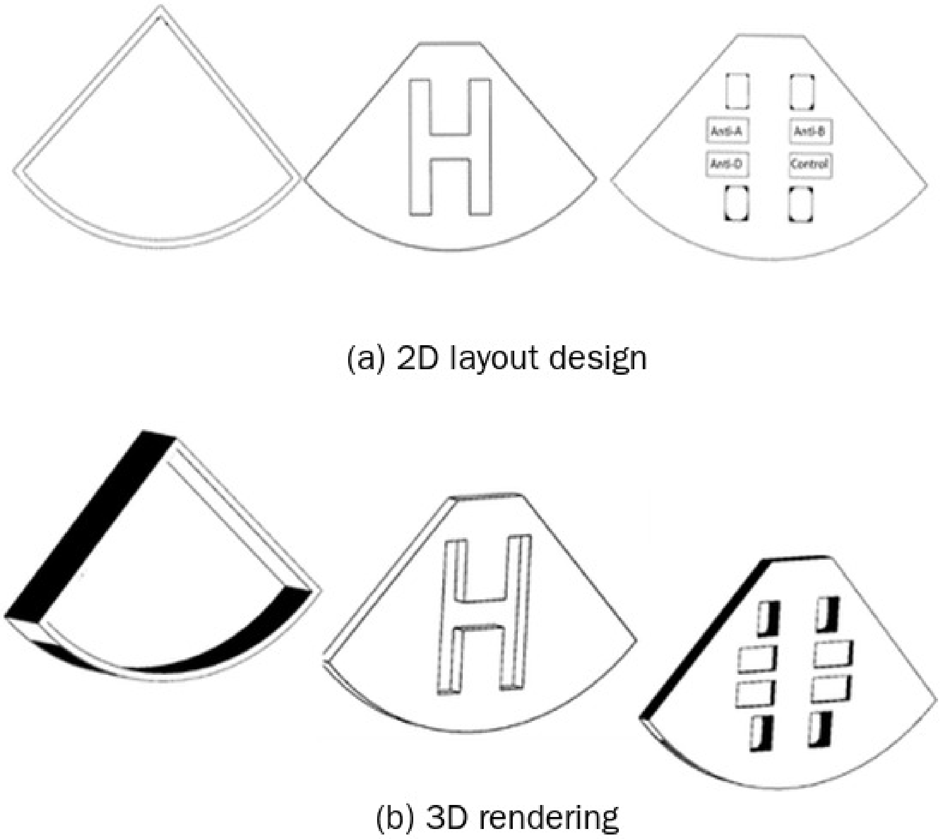


**Figure. S6.** Design of the fan-shaped device: (a) two-dimensional layout showing the fan outline, the H-shaped barrier and the four reaction zones (anti-A, anti-B, anti-D and control); (b) three-dimensional rendering of the two-compartment housing. Referenced in Section 3.4.

The specificity of antibody immobilisation was assessed functionally with the type-A blood-group test on Whatman No. 4 filter paper prepared under different surface conditions (Figure. S7). Each device carries four immobilised-antibody zones anti-A, anti-B, anti-D and a control. After application of the red

blood cells (RBCs) and the washing step, the distribution of cells retained within each detection zone was analysed with ImageJ to quantify RBC retention as an image-analysis signal. For type-A RBCs on GPTMS-functionalised paper, strong red signals appeared at the anti-A and anti-D zones consistent with correctly immobilised anti-A and anti-D antibodies (the sample being group A, RhD-positive), while the anti-B and control zones remained markedly fainter, demonstrating clear specificity for blood-group determination. In contrast, on the non-functionalised, APTES-functionalised and GA-treated papers the analysed signal was of similar magnitude across the anti-A, anti-B, anti-D and control zones, indicating non-specific retention with no discrimination between zones. These results further confirm that GPTMS-functionalised filter paper genuinely immobilises the antibodies and is suitable for ABO blood-group testing.

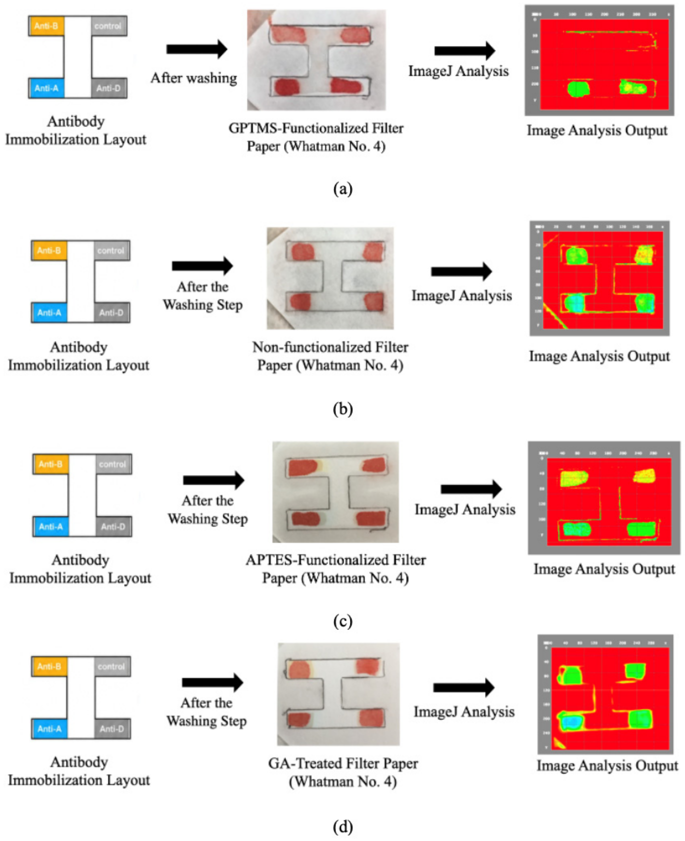


Figure. S7. Comparison of type-A RBC retention on differently treated Whatman No. 4 filter paper after washing. For each condition the antibody-immobilisation layout (anti-A, anti-B, anti-D and control), a representative device after the washing step, and the corresponding ImageJ image-analysis output are shown for (a) GPTMS-functionalised, (b) non-functionalised, (c) APTES-functionalised and (d) GA-treated filter paper. A specific pattern (anti-A and anti-D positive) is obtained only on the GPTMS route.

Microscale evidence of specific antibody–cell capture was obtained by scanning electron microscopy (SEM) of GPTMS-functionalised paper after the type-A RBC assay, using a cell-fixation protocol (Figure. S8). In the anti-A zone (positive reaction), numerous type-A red blood cells are captured and retained on the cellulose fibres, consistent with specific binding to the immobilised anti-A antibody. In the anti-B zone (negative reaction), very few cells remain after washing, because type-A cells are not recognised by anti-B and are removed by the wash. This microscale morphology complements the macroscopic ImageJ readout (Figure. S7): together they show that the antibodies immobilised on GPTMS-functionalised paper capture red cells specifically, confirming functional, covalent antibody immobilisation.

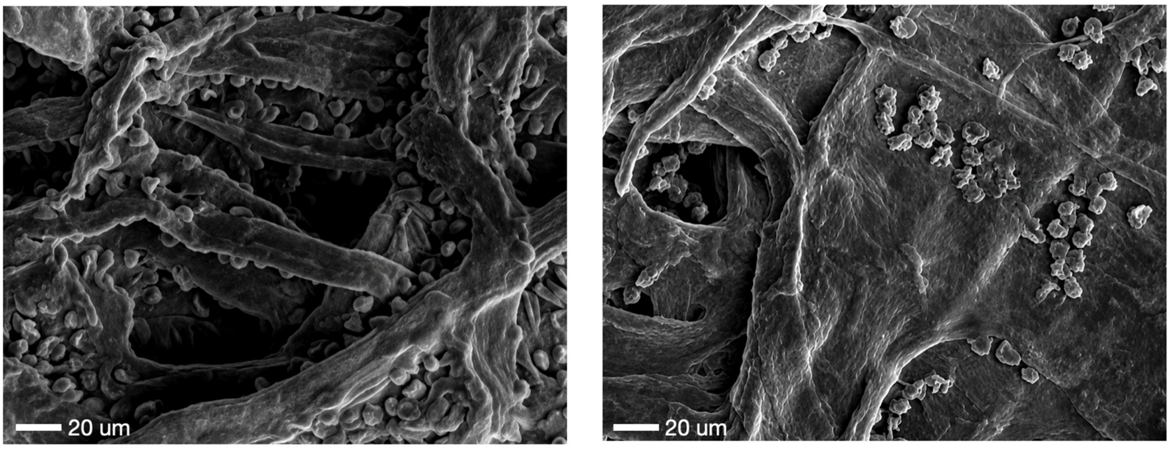


(a) (b)

Figure. S8. SEM images of GPTMS-functionalised filter paper showing the interaction of type-A red blood cells (RBCs) with immobilised antibodies using a cell-fixation method: (a) positive reaction of type-A RBCs in the anti-A zone and (b) negative reaction of type-A RBCs in the anti-B zone. Scale bars: 20 μm.

The direct consumable cost per test was estimated from bench-scale purchase prices and the measured amount of each material consumed per test. For each item, the per-test cost was calculated as the purchase price divided by the package quantity and multiplied by the usage per test; the individual contributions were then summed. On this basis the total direct consumable cost is approximately 11.6 THB (≈ 0.33 USD) per test. The largest contributions are the cellulose substrate and the disposable 3D-printed insert, which together account for roughly three-quarters of the total, while the antibody aliquots, wash buffer and other reagents each contribute a smaller share; the outer housing is reusable and is therefore not charged per test. These figures are laboratory-scale estimates based on current retail purchase prices and small-batch usage, and should be regarded as an upper bound. At manufacturing scale, bulk reagent

procurement, injection-moulded housings in place of FDM printing and automated dispensing would be expected to reduce the per-test cost substantially through economies of scale.
